# Neurodegeneration-associated protein VAPB regulates proliferation in medulloblastoma

**DOI:** 10.1038/s41598-023-45319-5

**Published:** 2023-11-09

**Authors:** Amanda Faria Assoni, Thiago Giove Mitsugi, René Wardenaar, Raiane Oliveira Ferreira, Elisa Helena Farias Jandrey, Gabriela Machado Novaes, Isabela Fonseca de Oliveira Granha, Petra Bakker, Carolini Kaid, Mayana Zatz, Floris Foijer, Oswaldo Keith Okamoto

**Affiliations:** 1 Human Genome and Stem Cell Research Center, Institute of Biosciences, University of São Paulo, 106, Rua do Matão, Cidade Universitária, São Paulo, 05508-090 Brazil; 2 European Research Institute for the Biology of Ageing, University of Groningen, 1, Antonius Deusinglaan, 9713 AV Groningen, The Netherlands

**Keywords:** Cancer, Cell biology, Molecular biology, Neuroscience

## Abstract

VAMP (Vesicle-associated membrane protein)-associated protein B and C (VAPB) has been widely studied in neurodegenerative diseases such as ALS, but little is known about its role in cancer. Medulloblastoma is a common brain malignancy in children and arises from undifferentiated cells during neuronal development. Therefore, medulloblastoma is an interesting model to investigate the possible relationship between VAPB and tumorigenesis. Here we demonstrate that high VAPB expression in medulloblastoma correlates with decreased overall patient survival. Consistent with this clinical correlation, we find that VAPB is required for normal proliferation rates of medulloblastoma cells in vitro and in vivo. Knockout of VAPB (VAPB^KO^) delayed cell cycle progression. Furthermore, transcript levels of WNT-related proteins were decreased in the VAPB^KO^. We conclude that VAPB is required for proliferation of medulloblastoma cells, thus revealing VAPB as a potential therapeutic target for medulloblastoma treatment.

## Introduction

Neurodegenerative diseases and cancer are very different pathologies, but a growing body of evidence is suggesting that both share deregulated mechanisms. Moreover, each are highly debilitating diseases for which few good treatment alternatives are available^1^.

Neurodegenerative diseases occur less frequently in cancer survivors and vice versa^2^. While this inverse correlation is well-established for neurodegenerative conditions such as Alzheimer’s and Parkinson’s disease, there is no clear consensus on this for amyotrophic lateral sclerosis (ALS)^3–5^.

ALS is the most frequent late-onset motor neuron disease and is characterized by the death of upper and lower glutamatergic motor neurons. Mutations in more than 40 different genes have been correlated with an increased risk of ALS development^6^. However, only a few overall deregulated genes have so far been described in different familial ALS and sporadic ALS. The VAMP (Vesicle-associated membrane protein)-associated protein B and C (*VAPB*) is one example of a gene differentially expressed independently of the specific mutations in sporadic ALS. Indeed, in some sporadic ALS patients, *VAPB* mRNA levels were found to be decreased in the spinal cord compared to healthy controls^7^. Furthermore, *VAPB* gene is associated with one of the familial forms of ALS, in which a single missense mutation causes motor neuron degeneration that leads to the phenotype of ALS8^8,9^. In addition to familial and sporadic ALS, *VAPB* differential expression has also been reported in cancer. A genome-wide microarray analysis of 50 human breast cancer cell lines and 145 clinical specimens revealed that *VAPB* is often amplified and/or overexpressed in breast cancer^10,11^.

A possible role for VAPB in cancer and ALS is further substantiated by the fact that VAPB interacts with Ephrin receptors^12^, which are key players in the development of the Central Nervous System (CNS) as well as in adult tissue homeostasis and which make up a large family of tyrosine kinase receptors^13^ that have been associated with both cancer and ALS^14^. For example, the EPH receptor A4(EPHA4) is a neural stem cells (NSC)-specific^15^ regulator of neurogenesis that maintains NSCs in an undifferentiated state and is furthermore expressed aberrantly in cancer cells, increasing tumor aggressiveness^16^.

These findings support a possible mechanism by which VAPB can influence both neurodegeneration and tumor development, particularly in the CNS.

In this study, we explore the potential role of VAPB in the context of cancer using medulloblastoma cell lines.

Medulloblastoma (MB) stands as the most prevalent malignant pediatric brain tumor, constituting approximately a quarter of all intracranial neoplasms^17^. Typically affecting children with a median age of 9 years and displaying a peak incidence between 3 and 7 years of age, MB represents a formidable challenge in pediatric oncology^18^. While the 5-year overall survival rate for MB is approximately 75%, the concern over long-term therapy-related morbidity remains a pivotal consideration. MB is inherently heterogeneous and is categorized into distinct subgroups including Wingless (WNT) MB, Sonic Hedgehog (SHH) MB, group 3 (G3—with commonly *c-MYC* alterations) MB and group 4 (G4) MB^19^. These molecular stratifications underscore the diverse nature of MB, necessitating a comprehensive understanding of its distinct molecular drivers and origins for the development of effective therapeutic strategies.

Medulloblastoma, arising from rhombic lip progenitors (WNT subtype), granule cell progenitors (SHH subtype), and unipolar brush cell progenitors (Groups 3 and 4) during early neural development^20^, enables investigating the functions of genes associated with both neurodegenerative conditions and malignancies. Despite the distinct origins of ALS-affected cells and medulloblastoma cells, they share the commonality of originating from neural cells. Furthermore, the constrained therapeutic options for medulloblastoma, underscore the significance of exploring novel pathways that could offer potential therapeutic avenues.

VAPB, a protein expressed ubiquitously, is particularly prominent in motor neurons and large neurons within the cerebellar nuclei. Conversely, its presence in neurons of the cerebellar cortex, including cerebellar Purkinje cells, exhibits comparatively lower levels^21^. The remarkable versatility of VAPB across diverse cellular contexts, demonstrated by its role in driving the proliferation of breast cancer cells, lends additional weight to its examination within the framework of medulloblastoma. As such, we leverage medulloblastoma cell lines as a tool to investigate the potential intersections between VAPB and pathways that hold relevance for both neurodevelopment and the progression of cancer^20^.

## Materials and methods

### Cell lines and cultures

Two embryonal CNS tumor cell lines, namely DAOY and USP13-Med, were utilized in this study. The DAOY cell line, derived from medulloblastoma, was obtained from ATCC (RRID:CVCL_1167; ATCC HTB-186), and its growth conditions followed ATCC recommendations. High-resolution karyotype analysis was performed to authenticate the DAOY cells. The USP13-Med cell line, also derived from medulloblastoma, was isolated and characterized as previously described^22^. To serve as a control, a human-induced pluripotent stem cell (hiPSC) line designated C2535, which was reprogrammed in-house, was utilized. Additionally, neural progenitor cells (NPCs) derived from hiPSCs were generated and characterized following the methodology described in Oliveira et al.^23^. All cell lines, including the DAOY, USP13-Med, hiPSCs, and NPCs, were cultured under standard conditions at 37 °C with 5% CO2 for up to four weeks (passages 1–4). Prior to their use in the experiments, all cell lines were subjected to PCR analysis (using the MP002 kit from Sigma-Aldrich) to confirm the absence of Mycoplasma contamination.

### Impact of *VAPB* expression on the survival of patients with medulloblastomas

Clinical information pertaining to overall survival and microarray expression data from a cohort of 632 medulloblastoma patients was acquired from the Cavalli dataset. To analyze and visualize the data, the Gliovis portal was utilized^24^. The patients were categorized into two groups based on their *VAPB* expression levels in the dataset: *VAPB*low (corresponding to the 25th quartile) and *VAPB*high (corresponding to the 75th quartile). The survival rates of these patient groups were compared using a Kaplan–Meier curve, and statistical analysis was performed using the log-rank test.

### Immunofluorescence

The cells were subjected to two washes, followed by fixation in 3.7% formaldehyde at room temperature for 20 min. To enable permeabilization, a solution containing 0.1% Triton X-100 and 5% bovine serum albumin in 1 × PBS was applied for 30 min. Subsequently, the cells were incubated overnight at 4 °C with the appropriate primary antibody (refer to the Antibody table). After three washes with 1 × PBS, the cells were incubated with the corresponding secondary antibody for 45 min (refer to the Antibody table). Following an additional two washes with 1 × PBS, the cell nuclei were stained with 1 μg/mL DAPI for 2 min. Finally, the cells were mounted on glass slides and cover-slipped using VectaShield mounting medium. All imaging was performed using a confocal microscope (Zeiss LSM 800).

### Experiments design and statistical analysis

All experimental procedures were conducted using biological triplicates or more replicates, as indicated in the figures. Furthermore, each experiment was independently performed three times. No blinding was implemented in the study, and there were no exclusions from the analysis. Statistical analysis was carried out using the Kruskal–Wallis test, followed by the Bonferroni post hoc test for multiple comparisons. For pairwise comparisons, a two-tailed unpaired *t* test was employed. The Fisher exact test was utilized to analyze clinical and pathological parameters. GraphPad Prism software (version 6.0, GraphPad Software Inc.) was employed for all statistical analyses. Quantitative data are presented as mean ± standard deviation (SD), and statistical significance was determined using the following thresholds: *p < 0.05; **p < 0.01; ***p < 0.001; and ****p < 0.0001.

### RNA sequencing

Following RNA extraction, the quality and quantity of total RNA were evaluated using a Nano chip on a 2100 Bioanalyzer (Agilent, Santa Clara, CA) according to the manufacturer’s instructions (Qiagen). Only total RNA samples with RNA Integrity Number (RIN) scores greater than 8 were included for library generation. For library construction, 250–500 ng of total RNA was used as input for mRNA enrichment using NEXTflex Poly(A) Beads (Bio Scientific) followed by library preparation using a NEXTflex Rapid Directional qRNA-Seq Kit (Bio Scientific). following the manufacturer’s instructions.

Paired-end sequencing was performed using a NextSeq 500 machine (Illumina; read 1 up to 74 cycles and read 2 up to 9 cycles). Read 1 contained the first STL (stochastic labeling) barcode, followed by the first bases of the sequenced fragments, and read 2 only contained the second STL barcode. The generated data were subsequently demultiplexed using sample-specific barcodes and changed into fastq files using bcl2fastq (Illumina; version 1.8.4).

The quality of the data was assessed using FastQC (Andrews, 2010^25^). The STL barcodes of the first read were separated from the sequenced fragments using an in-house Perl script. Low quality bases and parts of adapter sequences were removed with Cutadapt (Martin 2011^26^) (version 1.12; settings: q = 15, O = 5, e = 0.1, m = 36). Sequenced poly A tails were removed as well, by using a poly T sequence as adapter sequence (T{100}; reverse complement after sequencing). Reads shorter than 36 bases were discarded. The trimmed fragment sequences were subsequently aligned to all known human cDNA sequences (Ensembl cdna and ncrna; GRCh38 release 101) using HISAT2 (Kim et al. 2015^27^) (version 2.1.0; settings: k = 1000, –norc). The number of reported alignments, k, was given a high number in order to not miss any alignment results. Reads were only mapped to the forward strand (directional sequencing). Fragment sequences that mapped to multiple genes were removed (unknown origin). When fragments mapped to multiple transcripts from the same gene all but one were given a non-primary alignment flag by HISAT2 (flag 256). These flags were removed (subtraction of 256) by the same Perl script in order to be able to use the Bash-based shell script (dqRNASeq; see below) that is provided by Bioo Scientific (Perkin Elmer, MA, USA). Fragments that mapped to multiple transcripts from the same gene were considered unique and were counted for each of the transcripts. The number of unique fragments (or read pairs) was determined for each transcript using the script provided by Bio Scientific (dqRNASeq; settings: s = 8, q = 0, m = 1). This script uses as input the alignment results (bam file) as well as the two fastq files with only the STL barcodes (separated before from the sequenced fragments). Counts that were used for further analysis were based on a unique combination of start and stop positions and barcodes (USS + STL).

Differential gene expression analyses was performed R (version 3.6.3) using the R package DESeq2^28^ (version 1.26.0) using default settings (Negative Binomial GLM fitting and Wald tatistics), following standard normalization procedures. For all comparisons a simple design was implemented (design =  ~ condition-treatment). Pathway enrichment analyses were performed using the significantly differentially expressed genes using Enrichr^29^. Data is available in ArrayExpress under accession ID E-MTAB-11914.

### Edu labeling

Tumor cells were treated with a 10 µM concentration of EdU (5-ethynyl-2’-deoxyuridine) using the Click-It EdU Alexa Fluor 488 Imaging Kit (Life Technologies) for a duration of 30 min. Following the EdU incubation, the cell nuclei were stained with DAPI at a concentration of 5 µg/ml for 5 min. Confocal microscopy (Zeiss LSM 800) was employed to capture images of the stained cells, which were subsequently subjected to particle automated analysis using ImageJ software.

### 3D tumor spheroid assay

To induce tumor sphere formation, cells were seeded in a 96-well ultra-low attachment plate at an initial density of 1500 cells/ml. The culture medium used was DMEM/F12 supplemented with B-27, N-2, 20 ng/ml EGF, and 20 ng/ml bFGF (Invitrogen, Carlsbad, CA, USA). The plates were then incubated at 37 °C with a 5% CO_2_ humidified atmosphere for either 4 or 7 days, as specified in each respective figure. After the designated incubation period, the tumor spheres were dissociated for 20 min using Tryple at 37 °C. The dissociated cells were then centrifuged and counted using trypan blue staining to determine cell viability.

### Cell population growth assay

For the evaluation of proliferation rates in the adherent cell population, 500,000 cells were seeded in triplicate per well on day 0 in 6-well plates. Subsequently, the cells were detached and counted using the automated Countess system (Invitrogen) every other day or as specified in the corresponding figure.

### Plasmids

The generation of VAPB^KO^ cell lines was accomplished using CRISPR genome engineering. To target exon 2 of the VAPB gene, three guide RNAs were designed utilizing the guide resource tool from the Zhang lab^30^. These guide RNAs were subsequently cloned into the lenti-CRISPR plasmid, following the protocol outlined in reference^31^. The guideRNA that produced the highest number of knockout clones was selected for further experiments, with the forward sequence being “caccgTGAAGACTACAGCACCACGT” and the reverse sequence “aaacACGTGGTGCTGTAGTCTTCAc”.

To establish a doxycycline-inducible expression system for HA-VAPB overexpression, the full-length cDNA encoding VAPB was inserted between the BamHI and EcoRI sites of the retroviral plasmid pRetroX-Tight-BlastR, with the puromycin resistance gene replaced by blasticidin.

For transient expression, the pEGFPC1-hVAP-B plasmid, kindly provided by Catherine Tomasetto (Addgene plasmid #104448), was utilized. Additionally, the FUCCI (Fluorescent Ubiquitination-based Cell Cycle Indicator) plasmids were derived from the publication by Sakaue-Sawano et al.^32^.

### Time-lapse imaging

Time-lapse imaging was conducted using a DeltaVision microscope (Applied Precision Ltd./GE) to monitor cellular dynamics. For this, a population of 50,000 cells expressing the FUCCI system was initially seeded in 4-well imaging chambers (LabTech). Subsequently, images were acquired at 7-min intervals using a 20X objective lens. The analysis of cell cycle changes was performed, partially through manual examination, utilizing the softWoRxExplorer software (Applied Precision Ltd./GE).

### Western blot

Cells were collected through trypsinization and subsequently lysed using elution buffer (150 mM NaCl, 0.1% NP-40, 5 mM EDTA, 50 mM HEPES pH 7.5), supplemented with complete protease inhibitor (Roche), at 4 °C for 15 min. The lysates were then centrifuged at 300 g and 4 °C for 10 min to remove insoluble residues. For protein analysis, 20 µg of each sample was loaded onto 10% polyacrylamide gels. The proteins were transferred onto polyvinylidene difluoride (PVDF) membranes. Following transfer, the membranes were blocked in Odyssey blocking buffer (Li-cor Biosciences) at 4 °C for 60 min. Subsequently, the membranes were incubated overnight at 4 °C with the primary antibody (refer to Supplementary Table 2 for details). After incubation, the membranes were washed three times with 1 × PBS containing 0.1% Tween 20 (Sigma) and incubated with the corresponding secondary antibody for 1 h at room temperature. Blots were visualized using the Odyssey imaging system (Li-cor Biosciences), and the levels of protein expression were quantified using Image Studio Lite software (Li-cor Biosciences). In Fig. 4D, full length membrane is not available on our supplementary material “western blots” due to the automatic gel imaging, which identified membrane size and acquired an image of only the membrane area, without edges.

### Co-immunoprecipitation

Co-immunoprecipitation of HA-VAPB from medulloblastoma cell lines was performed using the Dynabeads Protein A/G Immunoprecipitation Kit (ThermoFisher). The protocol provided by the manufacturer was followed. Initially, 10 μL of monoclonal anti-HA antibody (Sigma) or 10 μL of mouse IgG (Santa Cruz Biotechnology) was added to create the Co-IP bead complex. The cells were washed once with PBS and then lysed in 500 µL of lysis buffer per well. The lysis buffer contained 20 mM Tris pH 8.0, 10% glycerol, 135 mM NaCl, 0.5% NP-40, and protease inhibitors (Complete, EDTA-free, Roche). The cell lysates were incubated on ice for 15 min and then centrifuged at 16,100 g for 5 min at 4 °C to remove cell debris. The input was mixed with the prepared beads that had been equilibrated in wash buffer and incubated for 1 h at 4 °C. After three washes with wash buffer, the beads were resuspended in RapiGest (Waters) and incubated at 65 °C for 10 min.

### In vivo animal study

The orthotopic model of embryonal CNS tumors was conducted using DAOY control (6 animals) and DAOY VAPB^KO^ (6 animals), as well as USP13-Med control (7 animals) and USP13-Med VAPB^KO^ (7 animals), following the previously described method^33^. Each individual animal served as an experimental unit, and all animals were included in the analysis. Random assignment of mice to groups ensured there were no significant differences in body weight and age between the groups. Anesthesia was administered using ketamine:xylazine at a dose of 200/15 mg/kg.

The surgical procedure involved injecting a tumor cell suspension (10^6^ cells in 5 μL of DMEM) into the right lateral ventricle of 3-month-old female Balb/C nude mice using a high-precision microsyringe (701RN; Hamilton Company, Reno, NV, USA) at a rate of 1 μL/min during stereotaxic surgery. The animals' weights were monitored every 2 days until weight loss exceeded 30% and/or visible tumor formation and/or ataxia were observed, at which point they were euthanized. If none of these symptoms were identified within 175 days after cell inoculation, the animals were euthanized to conclude the experiment. All necessary measures were taken to minimize animal suffering in accordance with the International Ethical Guidelines for Biomedical Research (CIOMS/OMS, 1985). Mice were euthanized by carbon dioxide asphyxiation (CO_2_) inhalation. This preclinical study adhered to the Animal Research: Reporting of In Vivo Experiments (ARRIVE guidelines 2.0) and was approved by the Institutional Animal Experimentation Ethics Committee of the Bioscience Institute, University of São Paulo (CEUA 291/2017).

### Ethics approval

This study followed the International Ethical Guideline for Biomedical Research (CIOMS/OMS, 1985) and was approved by the Institutional Animal Experimentation Ethics Committee of Bioscience Institute from University of São Paulo (CEUA 291/2017).

## Results

### Inactivation of VAPB in medulloblastoma cell lines decreases cell proliferation

Identified as downregulated in amyotrophic lateral sclerosis (ALS)^9^ and upregulated in breast cancer^10^, VAPB emerges as a protein with intriguing associations in distinct disease contexts. These observations prompted our investigation into the potential role of VAPB in maintaining cell viability and proliferation. In our pursuit to elucidate the implications of VAPB, we turned our focus to medulloblastoma – a suitable choice due to its origins from neural progenitor cells, its scarcity of safe and effective treatments, and the compelling need to unravel novel pathways for potential therapeutic avenues. To explore VAPB function in medulloblastoma, we employed two distinct medulloblastoma cell lines. The DAOY cell line, representative of the Sonic Hedgehog (SHH) subgroup, and the USP13-Med cell line, which was obtained from a 3-year-old boy with classic medulloblastoma, subgroup 4^22^. We generated VAPB knockout cell lines (KOs) using CRISPR-Cas9 system and isolated VAPB^KO^ single-cell clones^22^. We also engineered control cell lines by expressing Cas9, without guide RNAs followed by isolation of single cell clones as done for the VAPB^KO^ cell lines. The resulting 12 clonal cell lines were used for all further experiments.

Efforts to establish single-cell clones from D283-Med and CHLA-01-MED cell lines were made; however, these attempts were unsuccessful as the cells did not continue to proliferate after single-cell clone isolation, thereby limiting the generation of stable single-cell lines.

**Figure 1:**
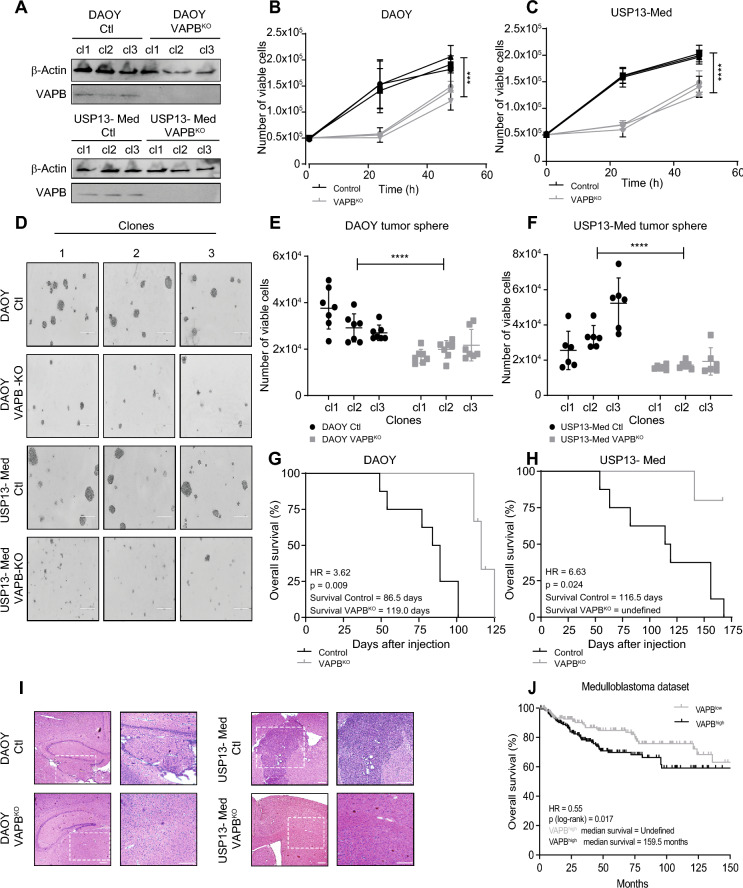
VAPB knockout decreases cell proliferation in 2D and 3D cultures of medulloblastoma cell lines. (**A**) Western blot against VAPB and β-actin of 3 single cell clones of DAOY and USP13-Med wildtype and VAPB^KO^ cell lines. (**B**) Cell count of 3 single cell clones of DAOY VAPB^KO^ and controls at 24 h and 48 h (linear fit curve test, p = 0.0016; n = 3). (**C**) Cell count of 3 single cell clones of USP13-Med VAPB^KO^ and controls at 24 h and 48 h (linear fit curve test, p = 0.0014; n = 3). (**D**) Representative phase contrast images of tumor spheres of DAOY and USP13-Med wildtype and VAPB^KO^ at 144 h post cell plating. Scale bar, 400 μm. (**E**) Quantification of the number of viable cells after dissociation of tumor spheres of 3 single cell clones of DAOY VAPB^KO^ and controls at 144 h post cell plating (Kruskal Wallis test, p = 0.0001; n = 3). (**F**) Quantification of the number of viable cells after dissociation of tumor spheres of 3 single cell clones of USP13-Med VAPB^KO^ and controls at 144 h post cell plating (Kruskal Wallis test, p = 0.0001; n = 3). (**G**) Survival curve of nude mice bearing DAOY VAPB^KO^ and control tumors (Log-rank test, p = 0.009; n = 6 per group). (**H**) Survival curve of nude mice bearing USP13-Med VAPB^KO^ and control tumors (Log-rank test, p = 0.02; n = 7 per group). (**I)** Representative phase contrast H&E images of euthanized mice brain bearing DAOY and USP13-Med wildtype and VAPB^KO^ tumors. Scale bar, 400 μm. (**J**) Impact of *VAPB low* or *high* expression on the survival of patients with medulloblastomas in Cavalli’s dataset (Log-rank test, p = 0.008; n = 317).

Loss of VAPB protein expression was confirmed by western blot for each single cell clone (Fig. 1A). We noted that VAPB^KO^ clones in the USP13-Med line displayed a remarkably altered morphology, as they became more flattened and more elongated (Sup. Fig. 1A). DAOY VAPB^KO^ cells did not show morphological changes.

Furthermore, USP13-Med and DAOY VAPB^KO^ cells showed decreased proliferation rates compared to controls (Fig. 1B and C). Cell growth experimental procedures were carried out using 24-well plates, where both wild-type and VAPB^KO^ clones were seeded at a consistent confluence of 50,000 cells per well. We acknowledge that the growth curve of wild-type clones exhibited a steep slope in the first 24 h, however, the growth curve slope in the 24–48 h period was more moderate. This is probably due to the Control clones reaching almost full confluence by the 48-h time point (Supplementary Fig. 1F), which did not occur in cultures of VAPB^KO^ cells. Notably, the delayed onset of cell cycling in VAPB^KO^ cells after passaging allowed them to continue proliferating during the experimental time frame, thereby impacting the overall growth dynamics.

We further assessed tumorigenic potential of VAPB^KO^ cells in a 3D model, using a sphere formation assay (SFA). We found that VAPB^KO^ cells were less capable of forming spheres (Fig. 1D–F; and Sup. Fig. 1B–E).

We next set out to validate these findings in vivo*.* To test tumorigenicity of the cells in their origin tissue, we performed orthotopic xenografting experiments in BALB/c nude mice. In this setting, both DAOY as well as USP13-Med cell lines formed tumors in vivo and overall survival is displayed in Fig. 1G and H. Histologic analysis of the brain of terminated mice confirmed tumors in brain tissue in every group (Fig. 1I), although VAPB^KO^ tumors were smaller.

To determine the relevance of our findings for human patients, we evaluated the impact of *VAPB* expression on the overall survival of medulloblastoma patients from Cavalli’s dataset^34^. In line with our previous findings, we found that increased *VAPB* expression (VAPB^high^) is associated with reduced overall medulloblastoma patient survival (Fig. 1J). However, the expression levels of VAPB might be distinct between medulloblastoma molecular subtypes. Therefore we evaluated VAPB expression in medulloblastoma patient samples according to their subtype, using Cavalli’s dataset. Our findings indicate that VAPB expression in G3 and G4 tumors is significantly higher than the expression levels in SHH tumors. However, no significant differences in VAPB expression levels were detected among tumors of the WNT, G3, and G4 subgroups. Also, when conducting survival analysis in separate subgroups (Supplementary Fig. 2), no statistically significant correlation was found between VAPB expression and overall survival within each of these molecular subgroups. Thus, the correlation of high VAPB expression with shorter overall survival of medulloblastoma patients presented in Fig. 1J does not seem to depend on the specific tumor molecular subtype. Furthermore, we conducted an analysis of VAPB expression in available datasets, comparing tumor tissue with non-tumor tissue (Supplementary Fig. 2F).

We conclude that *VAPB* expression is an important parameter for medulloblastoma growth, particularly in vitro and, therefore, further explored VAPB^KO^ cells to better understand the molecular role of VAPB in medulloblastoma biology.

### Reconstitution of VAPB expression in VAPB^KO^ medulloblastoma cells restores cell proliferation in a dose-dependent fashion

**Figure 2:**
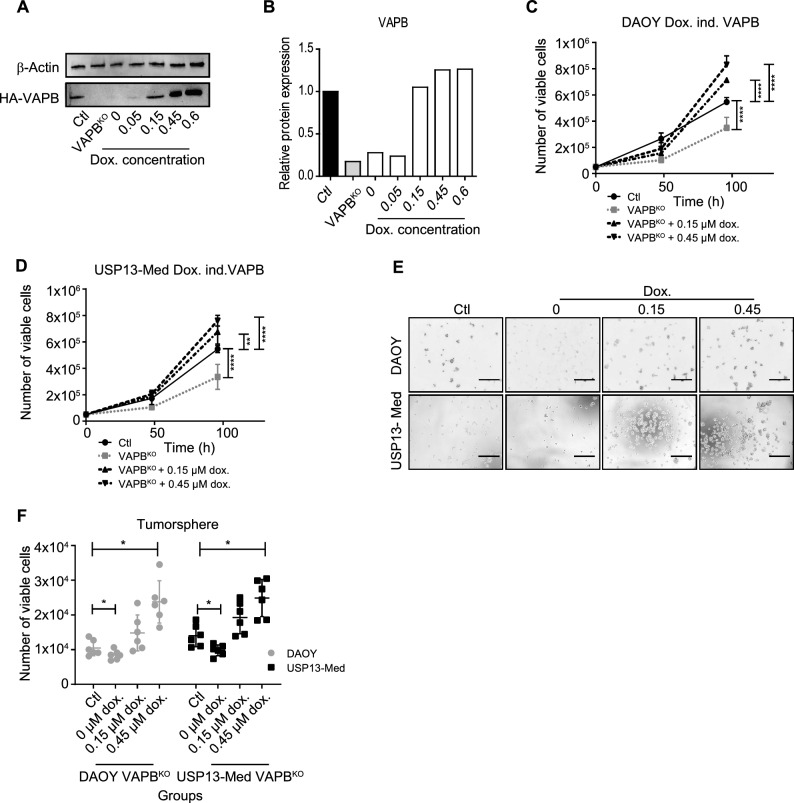
VAPB re-expression restores proliferation and tumor sphere formation ability in medulloblastoma cell lines in a dose-dependent manner. (**A**) Representative western blot for HA-VAPB and β-actin on one VAPB^KO^ clone with doxycycline-inducible HA-tagged VAPB with addition of different concentrations of doxycycline: 0 µM, 0.05 µM, 0.15 µM, 0.45 µM and 0.6 µM. (**B**) Quantification of the corresponding densitometry of VAPB protein bands of one VAPB^KO^ clone with doxycycline-inducible HA-tagged VAPB with addition of different concentrations of doxycycline: 0 µM, 0.05 µM, 0.15 µM, 0.45 µM and 0.6 µM. (**C**) Cell count of 3 single cell clones of DAOY controls and VAPB^KO^ with doxycycline-inducible HA-tagged VAPB at 48 h and 96 h after addition of different concentrations of doxycycline (Kruskal Wallis test with Bonferroni multiple comparisons corrections; n = 3) (**D**) Cell count of 3 single cell clones of USP13-Med controls and VAPB^KO^ with doxycycline-inducible HA-tagged VAPB at 48 h and 96 h after addition of doxycycline (0.45 µM) (Kruskal Wallis test with Bonferroni multiple comparisons corrections; n = 3). (**E**) Representative phase contrast images of tumor spheres of one DAOY and one USP13-Med clone with Tet-On HA-tagged and different concentrations of doxycycline at 48 h hours post cell plating. Scale bar, 400 μm. (**F**) Quantification of the number of viable cells after dissociation of tumor spheres at 96 h post cell plating and addition of different doxycycline concentrations of 3 single cell clones of DAOY and USP13-Med controls and VAPB^KO^ (Kruskal Wallis test with Bonferroni multiple comparisons corrections; n = 6 per group).

To confirm whether the reconstitution of VAPB expression would recover the proliferation of cultured VAPB^KO^ medulloblastoma cells, we overexpressed HA-tagged VAPB under control of a Tet-On (i.e. doxycycline-sensitive) promoter. We added increasing concentrations of doxycycline (0.15–0.45 µM) to the culture media and confirmed restoration of VAPB protein expression by assessing the levels of HA-VAPB by western blot (Fig. 2A and B).

Indeed, we found that reconstituted VAPB expression significantly recovered the in vitro proliferation rates of the tumor cells (Fig. 2C and D) as well as their ability of generating spheres (Fig. 2E and F). On top of that, the cell proliferation rates were dependent on the expression levels of VAPB: the more VAPB protein present, the higher the proliferation rate (Fig. 2B–F), underscoring the relevance of VAPB protein for the in vitro expansion of medulloblastoma cells.

In order to assess any potential impact of doxycycline on cell proliferation, we conducted additional experiments applying doxycycline in DAOY and USP13-Med wild-type and VAPB^KO^ cells. Growth curves were generated for these cells without or with 0.45 µM doxycycline. The results, as depicted in Supplementary Fig. 2G and H, reveal that there are no significant differences in growth kinetics among the tested conditions. This indicates that doxycycline administration at the specified concentration did not exert discernible effects on cell proliferation. Given the consistency of these findings with our previous results, we conclude that the observed effects of HA-VAPB inducible expression on cell growth are unlikely to be attributed to doxycycline administration.

To better understand how VAPB influences cell proliferation, we next investigated the consequences of VAPB absence on cell cycle dynamics of our medulloblastoma lines.

### VAPB^KO^ causes cell cycle arrest in G0/G1

**Figure 3:**
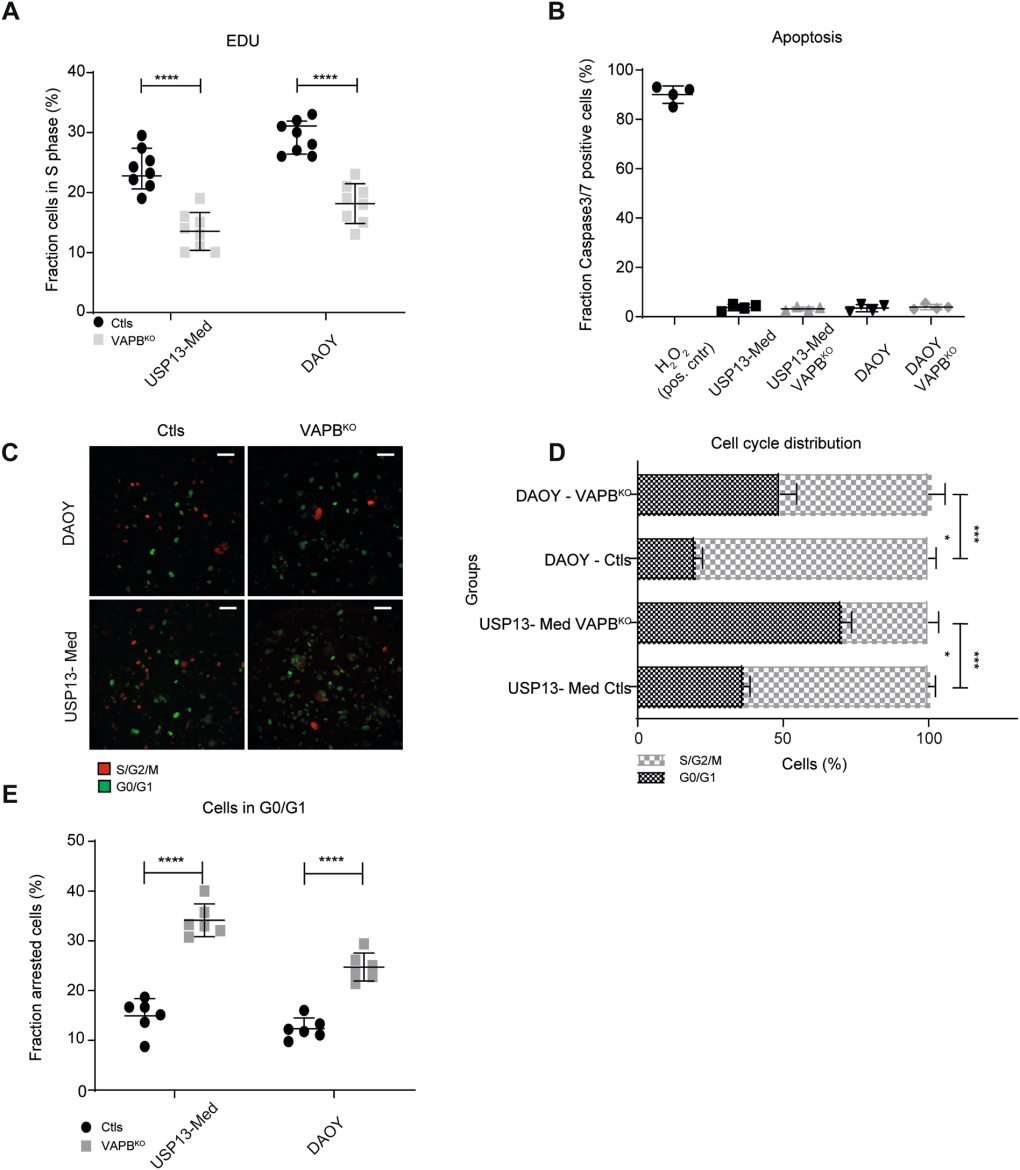
VAPB^KO^ causes cell cycle delay in G0/G1. (**A**) Quantification of tumor cell proliferation based on EdU incorporation of 2 single cell clones per group of DAOY and USP13-Med controls and VAPB^KO^ cells (Kruskal Wallis test with Bonferroni multiple comparisons corrections; n = 8). (**B**) Quantification of the number of cells with active Caspase 3/7 on a positive control treated with H_2_O_2_ for 24 h and on the DAOY and USP13-Med single cell clone controls and VAPB^KO^ (Kruskal Wallis test with Bonferroni multiple comparisons corrections; n = 4). (**C**) Representative images of time-lapse imaging of DAOY and USP13-Med single cell-derived clones of VAPB^KO^ and controls expressing the FUCCI system with GFP-cdt1 and RFP-geminin. Scale bar, 25 μm. (**D**) Quantification cell cycle distribution (G1/G0 or S/G2/M) of DAOY and USP13-Med single cell clones of VAPB^KO^ and controls (Kruskal Wallis test with Bonferroni multiple comparisons corrections; n = 4). (**E**) Quantification of the percentage of cells that remain in G1/G0 all throughout the length of the time-lapse imaging record (60 h) (Kruskal Wallis test with Bonferroni multiple comparisons corrections; n = 6).

Since VAPB is an endoplasmic reticulum (ER) protein that had not yet been related to the cell cycle, we performed additional cell cycle assays to better understand the decreased proliferation of cells lacking the VAPB protein. First, we observed by EdU (5-ethynyl-2’-deoxyuridine) incorporation that the VAPB^KO^ clones had fewer cells in S phase (Fig. 3A). Second, by quantifying the number of cells with cleaved Caspase 3/7, we found that the decreased growth of the VAPB^KO^ cells was not due to increased apoptosis (Fig. 3B). We therefore wanted to test whether VAPB loss would affect cell cycle dynamics. To test this, we introduced the FUCCI system into our VAPB cell lines. The FUCCI (Fluorescence Ubiquitin Cycle Indicator) system combines two cell cycle markers, GFP-tagged CDT1 to label cells in G0/G and RFP-tagged Geminin to label cells in G2, allowing to precisely quantify cell cycle dynamics by time-lapse imaging.

As shown in Fig. 3C–E, time-lapse imaging of FUCCI-labeled cell lines revealed that VAPB^KO^ cells accumulated in the G0/G1 phase of the cell cycle, suggesting that cells exhibited delayed cell cycle progression (Fig. 3C and D) and remained arrested throughout the experiment (60 h) (Fig. 3E).

### Medulloblastoma cell proliferation is restored by inhibition of EPHA4 phosphorylation in VAPB^KO^ cells

As VAPB is known to interact with Ephrin receptors^12,35^, we next investigated whether deregulation of EPHA4, a known VAPB interactor, contributed to the proliferation defect observed in VAPB^KO^ cells.

We first assessed the expression patterns of EPHA4 in medulloblastoma. For this, we took advantage of previously published datasets that characterized primary medulloblastoma samples^24^ and compared *EPHA4* gene expression between medulloblastoma and healthy tissues (Sup. Fig. 3A). We indeed found that *EPHA4* gene expression was significantly decreased in medulloblastoma samples compared to unaffected tissue in each data set.

To test the functional importance of this finding in primary tumor samples, we next inhibited EPHA4 activity in medulloblastoma cell lines by impairing EPHA4 phosphorylation using a KYL peptide^36^. Here we found that inhibition of EPHA4 phosphorylation induced proliferation of cells cultivated as tumor spheres and would thus be expected to promote tumor growth (Sup. Fig. 3B and C). From these data, we conclude that downregulation of EPH4A protein or inactivation of EPHA4 through inhibiting its phosphorylation both promote medulloblastoma growth in a 3D setting.

While previous work has shown that VAPB interacts with EPHA4^12^, its effect on the receptor activation is not well understood. Therefore, we next investigated the interaction between EPHA4 and VAPB.

To understand how EPHA4 and VAPB interact, we first determined whether VAPB and EPHA4 physically interact in untransformed CNS cells. To this end, we performed a proximity ligation (PLA) assay in primary, non-transformed neuronal progenitor cells (NPCs), derived from induced pluripotent stem cells, which revealed co-localization of VAPB and EPHA4 (Sup. Fig. 3D and E), suggesting a direct interaction. To test whether this interaction also exists in medulloblastoma cell lines, we used our previously generated medulloblastoma cell lines expressing HA-tagged VAPB and performed co-immunoprecipitation as well as PLA assays using HA and EPHA4 as bait. However, in these medulloblastoma, we failed to detect colocalization of VAPB and EPHA4 in the PLA assay, nor did we find evidence for a direct interaction between both proteins in co-immunoprecipitation assays (Sup. Fig. 3F–H). As we could not find evidence for a direct interaction between VAPB and EPHA4, in medulloblastoma cells, we then investigated whether the absence of VAPB would affect the expression or phosphorylation of EPHA4.

We next tested EPHA4 protein expression and phosphorylation in the VAPB^KO^ clones. While inactivation of VAPB did not change EPHA4 protein levels (Sup. Fig. 4A and C), we did observe that EPHA4 phosphorylation was significantly increased in VAPB^KO^ clones compared to control cells (Sup. Fig. 4A–D). To test the functional relevance of this observation, we again took advantage of the KYL peptide to inhibit EphA4 phosphorylation^36^ and determined the effect on the proliferation of VAPB^KO^ cells. While untreated VAPB^KO^ clones exhibited a significantly decreased amount of viable cells forming tumorspheres compared to the control group (Sup. Fig. 4G–I), no significant differences in viable sphere cells were detected when VAPB^KO^ clones were treated with the KYL peptide. While KYL peptide treatment clearly increased the number of viable cells in the VAPB^KO^ spheres and recovered VAPB^KO^ cells proliferation rates, it is important to note that proliferation rates of VAPB^WT^ cells were also increased by KYL (Sup. Fig. 4E–G), suggesting that VAPB regulates other targets in addition to EPHA4 that affect medulloblastoma cell proliferation.

To check for evidence if VAPB might influence EPHA4 expression, we once again made use of the Cavalli dataset to verify whether there is a correlation of *VAPB* and *EPHA4* mRNA expression, which we could not find (Supplementary Fig. 4J). Our findings highlight the necessity to further confirm a relation of VAPB protein expression and EPHA4 phosphorylation status in primary human medulloblastoma samples.

### VAPB modulates WNT/β-catenin pathway in medulloblastoma cells

**Figure 4:**
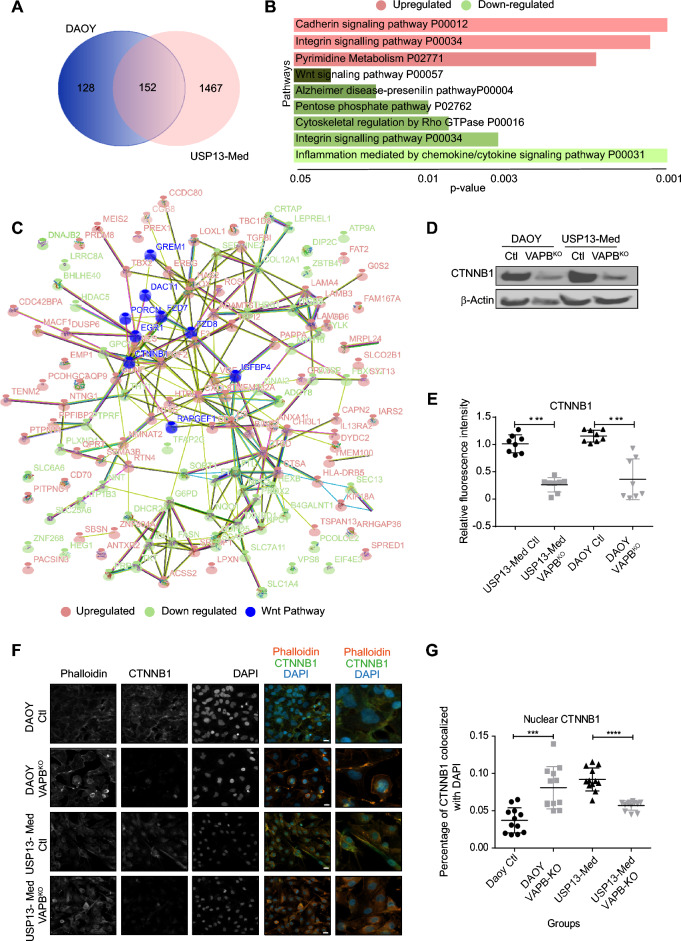
Loss of VAPB expression causes dysregulation of several signaling pathways. (**A**) Venn diagram of differentially-expressed genes in DAOY and USP13-Med VAPB^KO^ versus control cells. (**B**) Enriched pathways within differentially expressed genes for both DAOY and USP13-Med VAPB^KO^ cells. (**C**) Interactome mapping of differentially expressed genes in both DAOY and USP13-Med VAPB^KO^ cells, as identified by RNA sequencing. (**D**) Western blot of DAOY and USP13-Med single cell clones of VAPB^KO^ and controls showing total β-catenin. β-Actin is shown as a loading control. (**E**) Quantification of the relative fluorescence intensity of β-catenin from immunofluorescence stainings on DAOY and USP13-Med single cell-derived VAPB^KO^ and controls clones (Kruskal Wallis test with Bonferroni multiple comparisons corrections; n = 8). (**F**) Representative images of immunofluorescence stainings using anti-total β-catenin on DAOY and USP13-Med single cell-derived VAPB^KO^ and controls clones. (**G**) Quantification of the percentage of fluorescence intensity of β-catenin colocalized with DAPI from immunofluorescence stainings on DAOY and USP13-Med single cell-derived VAPB^KO^ and controls clones (Kruskal Wallis test with Bonferroni multiple comparisons corrections; n = 12).

To determine which other signaling cascades were affected in VAPB^KO^ cells, we next analyzed transcriptomes of VAPB^WT^ and VAPB^KO^ cells by RNA sequencing. We noted that the number of differentially expressed genes between VAPB^WT^ and VAPB^KO^ cells was much higher in the USP13-Med background than in DAOY cells (Fig. 4A). This finding agrees with the pronounced morphology change observed in USP13-Med VAPB^KO^s, an effect that was not detected in the DAOY VAPB^KO^ cells. Nonetheless, when analyzing the subset of differentially expressed genes in both cell lines, we found an overrepresentation of proliferation-related genes, including the WNT signaling pathway (Supplementary Table 1, Fig. 4B and C).

We therefore next assessed whether WNT signaling was indeed decreased in VAPB^KO^ cells and found by western blot and immunofluorescence that the CTNNB1 protein levels were indeed decreased in the VAPB^KO^s compared to controls (Fig. 4D–G).

While our study primarily focused on confirming the expression levels of the WNT signaling pathway due to its relevance to one of the medulloblastoma subtypes, we acknowledge the importance of considering other signaling pathways in the context of medulloblastoma biology. Specifically, integrin, cadherin, and cytoskeleton pathways have been recognized as potentially significant contributors to cellular phenotypes. We want to clarify that these pathways were not disregarded but were evaluated within the constraints of available resources. We recognize the value of investigating additional pathways and their potential interactions to gain a more comprehensive understanding of the complex regulatory networks underlying medulloblastoma progression.

## Discussion

Here we find that absence of VAPB decreases the proliferation potential of medulloblastoma cells in vivo and in vitro due to a cell-cycle delay. We also investigated the molecular pathways deregulated in medulloblastoma cells when VAPB is absent and observed that *VAPB*^*KO*^ in medulloblastoma cells results in increased phosphorylation of the EPHA4 receptor, as well as deregulated expression of WNT-regulated genes, both pathways with an established role in neurodevelopment^15,37,38^.

While direct physical interaction between VAPB and EPHA4 was not observed in our study, our RNA sequencing analysis of VAPB^KO^ clones of both DAOY and USP-13-Med cells revealed differential expression of downstream targets of EPHA4 activation, when compared to their respective wild-type clones. These include *FGF2*, IGF1-related genes, and *TGFβ*^16,40–41^*.* However, further studies are needed to fully understand if this phenomenon is relevant to medulloblastoma biology. For example, patient data accessing phosphorylation levels is essential to ascertain cell line data findings.

On the pathways differentially expressed between controls and VAPB^KO^, we found that VAPB^KO^ decreased WNT signaling. Indeed, WNT signaling is frequently affected in medulloblastoma development and one of the molecular subtypes of medulloblastoma is characterized by mutations in genes belonging to this pathway^42^. Furthermore, the *CTNNB1* gene is upregulated in most medulloblastoma samples^43,44^.

The role of WNT signaling in various contexts is complex and multifaceted^45–47^. In the case of medulloblastoma, while WNT pathway involvement is recognized, the WNT subgroup of tumors demonstrates a more favorable prognosis compared to tumors of other molecular subgroups, leading to suggestions of using WNT activation as a therapeutic strategy for non-WNT tumors^45,48,49^.

Importantly, we acknowledge that distinguishing the independent roles of EPHA4 and the WNT signaling pathway was not feasible within the scope of this investigation. Notably, prior literature has reported interactions between EPHA4 and CTNNB1^50,51^, adding an additional layer of complexity to their potential functional relationship. It will be critical to further evaluate whether VAPB could be involved in the control of EPHA4 activity and WNT signaling. In mammalian neurons, VAPB has been reported to interact with regulators of protein phosphorylation status^52^. Also, EPHA4 activity significantly increased the proliferation of neural stem cells by suppressing Wnt/β-catenin signaling^53^.

Even though further work is required to fully understand the relationship between VAPB, EPHA4, and WNT pathway regulation, the effect of VAPB inactivation on cell cycle progression can be well-explained by a combination of increased EPHA4 phosphorylation and decreased β-catenin levels – the main WNT pathway effector^54^– as found in the VAPB^KO^ clones. Possibly, the phenotypes observed for EPHA4 and WNT in VAPB^KO^ cells are related as EPHA4 was previously found to inhibit WNT signaling^55^. WNT signaling has a firmly established role in cell proliferation: β-catenin levels increase during S phase, reach maximum levels in late G2/M phase that then abruptly decrease when cells enter a new G1 phase^54^. This expression pattern allows a subset of WNT target genes to be transcribed specifically in both S and G2. Therefore, decreased β-catenin levels will impair cell cycle progression^54^.

## Conclusions

Our investigations revealed that VAPB depletion had robust effects on cell proliferation and cell cycle progression in vitro, underscoring its potential role in maintaining regular cell cycle dynamics. Our data suggest that VAPB functions to uphold proper cell cycle regulation, which could have notable implications for tumor growth. We acknowledge that medulloblastoma biology involves intricate interactions among numerous genes and pathways, each contributing to the complex nature of the disease. While we recognize the need for a comprehensive understanding of the multifaceted molecular landscape, our study contributes a significant piece to this intricate puzzle. By focusing on VAPB and its effects on cellular behavior, we provide a foundation for future investigations to unravel the mechanisms governing medulloblastoma and identify potential avenues for therapeutic interventions.

### Supplementary Information

Supplementary Information 1.

Supplementary Information 2.

## Data Availability

Most data generated or analysed during this study are included in this published article and its supplementary information files. The RNA sequencing raw data is available in ArrayExpress under acession ID E-MTAB-11914.The brain tumor expression datasets analysed during the current study are available in the GlioVis repository, [http://gliovis.bioinfo.cnio.es/]^20^.
