# Supplementary material for: Neurodegeneration-associated protein VAPB regulates proliferation in medulloblastoma
Source: Sci Rep. 2023 Nov 9;13:19481. doi: 10.1038/s41598-023-45319-5 (PMC10636017; doi:10.1038/s41598-023-45319-5)
Supplement: Supplementary file 1 — Supplementary Information 1. [file 41598_2023_45319_MOESM1_ESM.pdf]

Neurodegeneration-associated protein VAPB regulates proliferation in medulloblastoma

Amanda Faria Assoni[1,2] (0000-0001-9087-7823), Thiago Giove Mitsugi[1] (0000-0001-5940-194X), René Wardenaar[2], Raiane de Oliveira Ferreira[1] (0000-0003-2326-9494), Elisa H. F. Jandrey[1] (0000-0003-0750-7552), Gabriela Machado Novaes[1] (0000-0002-9699-5596), Isabela Fonseca de Oliveira Granha[1] (0000-0002-3180-3039), Petra Bakker[2] (0000-0001-7019-0258), Carolini Kaid[1] (0000-0002-1852-6997), Mayana Zatz[1] (0000-0003-3970-8025), Floris Foijer[2#*] (0000-0003-0989-3127), Oswaldo Keith Okamoto[1#*] (0000-0002-8528-6225).

1. Human Genome and Stem Cell Research Center, Institute of Biosciences, University of São Paulo, Cidade Universitária, São Paulo 055080-090, Brazil.

2. European Research Institute for the Biology of Ageing, University of Groningen, Groningen, 9713 AV, the Netherlands.

[#] These authors contribute equally

[*]to whom correspondence should be addressed:

Floris Foijer : 1, Antonius Deusinglaan, 9713 AV Groningen, f.foijer@umcg.nl, phone number: +31503617305;

Oswaldo Keith Okamoto: 106, Rua do Matão, 05508-090, São Paulo, keith.okamoto@usp.br phone number: +551130917966

Amanda Faria Assoni: 106, Rua do Matão, 05508-090, São Paulo, amandafassoni@gmail.com

phone number: +551130910878

Running title: VAPB and medulloblastoma cells proliferation

Key words: medulloblastoma; ALS; VAPB; proliferation; EPHA4; WNT

SUPPLEMENTARY MATERIAL

Supplementary table 1: List of differentially expressed genes in both DAOY and USP-13-Med VAPB^{KO} cells compared to controls. Green: Downregulated; Red: Upregulated.

Gene Names					
DIP2C	SORT1	DHCR24	LRRC8A	NMNAT2	TFAP2C
SLCO2B1	MEIS2	TKT	FAT2	FTL	IDI1
GDPD5	LINC02154	MACF1	TENM2	CORO1B	G6PD
TSPAN13	COPG1	LAMA2	CHI3L1	EMP1	THBS1
DYSF	QPRT	UTY	PAPPA	AC109635.5	TXNRD1
NPC2	SLC6A6	AQP9	LPXN	LAMB3	SLC25A6
HDAC5	CD6	HLA-DRB5	ANTXR2	IL13RA2	CD81
MRPL24	PRPS2	GNAI2	CXCL8	FTH1	RPL27A
C3	CDC42BPA	FASN	EGR1	PACSIN3	FZD8
SYT13	TBX2	B4GALNT1	LOXL1	FZD7	KIF18A
RAPGEF1	HAS2	ADCY8	RTN4	PTPRF	HTRA1
SEC13	NT5E	GREM1	CILK1	NQO1	BHLHE40
THY1	HEXB	BIRC2	PRDM8	ZNF268	CD70
TBC1D8	EREG	TMEM132A	MFAP4	MYLK	NTNG1
FAM167A	PTPN14	HEG1	CRABP2	CAPN2	EIF4E3
DYDC2	ZBTB47	CRTAP	DUSP6	SLC1A4	PRDX2
USP9Y	G0S2	VPS8	GDNF	P3H2	DNAJB2
MYH10	CTSD	PLXND1	SERPINE2	CCDC80	PPFIBP2
SPRED1	SEMA3B	TMEM100	ATP9A	PALMD	PORCN
CTNNB1	SLC7A11	ACSS2	ADAMTS1	FGF2	LOX
IARS2	PREX1	SREBF1	CGB8	TGFBI	ANXA11
ARHGAP36	CTSA	F3	TFPI2	ATP1B3	COL12A1
NES	VGF	IGFBP4	PCOLCE2	SCAP	SNHG29
NNT	SBSN	PITPNC1	SNHG14	NPC1	ROS1
PCDHGC3	LAMA4	ZNF804A	FBXO32	ITGB5	DACT1

Supplementary table 2: Antibody list

Brand	Antibody	Catalog number
Cell Signaling	beta-Actin (13E5) Rabb mAb	4970
ECM Biosciences	EPHA4 (Tyr-602)[conserved site], phospho-specific Antibody	EP2731
Santa cruz	anti-EPHA4	(sc-365503)
ProteinTech	VAPB	(66191-1-Ig)
Cell Signaling	CTNNB1	8480
ThermoFisher	Goat Anti-Mouse 594	A-11005
Thermo Fisher	Goat Anti-Rabbit 488	A-11008
Sigma	Anti-human IgG	**I6260**
Cell Signaling	HA-Tag (C29F4) Rabb mAb	3724

SUPPLEMENTAL FIGURES

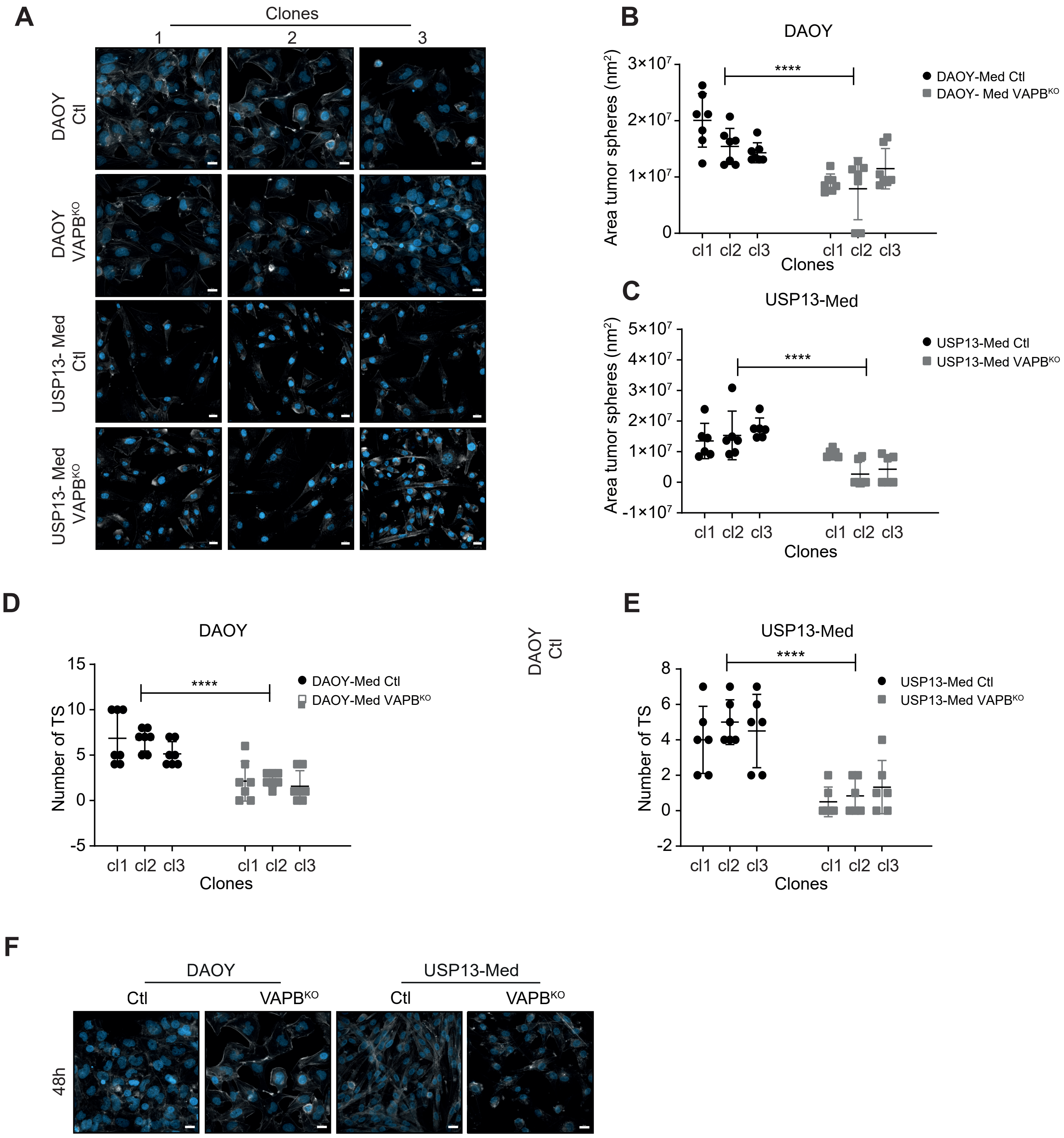
A
Clones
1
2
3
DAOY Ctl
DAOY VAPBKO
USP13- Med Ctl
USP13- Med VAPBKO
B
DAOY
Area tumor spheres (nm2)
3×107
2×107
1×107
0
****
DAOY-Med Ctl
DAOY- Med VAPBKO
cl1 cl2 cl3 cl1 cl2 cl3
Clones
C
USP13-Med
Area tumor spheres (nm2)
5×107
4×107
3×107
2×107
1×107
0
-1×107
****
USP13-Med Ctl
USP13-Med VAPBKO
cl1 cl2 cl3 cl1 cl2 cl3
Clones
D
DAOY
Number of TS
15
10
5
0
-5
****
DAOY-Med Ctl
DAOY-Med VAPBKO
cl1 cl2 cl3 cl1 cl2 cl3
Clones
DAOY Ctl
E
USP13-Med
Number of TS
8
6
4
2
0
-2
****
USP13-Med Ctl
USP13-Med VAPBKO
cl1 cl2 cl3 cl1 cl2 cl3
Clones
F
DAOY
USP13-Med
Ctl
VAPBKO
Ctl
VAPBKO
48h

Supplemental. 1: VAPB knock-out changes cell morphology, decreases cell proliferation *in vitro* and tumor growth *in vivo.* **a.** Representative phalloidin stainings of clones from DAOY and USP13-Med controls or VAPB^{KO} cells. Scale bar 20 µm. **b**. Quantification of the area of tumor spheres from DAOY controls or VAPB^{KO} cells at 144 h post cell plating (Kruskal Wallis test with Bonferroni correction; n = 7 per group). **c**. Quantification of the area of tumor spheres from USP13-Med controls or VAPB^{KO} cells at 144 h post cell plating (Kruskal Wallis test with Bonferroni correction; n = 7 per group). **d**. Quantification of the number of tumor spheres of tumor spheres from DAOY controls or VAPB^{KO} cells at 144 h post cell plating (Kruskal Wallis test with Bonferroni correction; n = 7 per group). **e.** Quantification of the number of tumor spheres from USP13-Med controls or VAPB^{KO} cells at 144 h post cell plating (Kruskal Wallis test with Bonferroni correction; n = 7 per group).**f**. Representative pictures of phalloidin stainings of one clone each from DAOY and USP13-Med controls or VAPB^{KO} cells 48h after plating for the growth curve experiment.

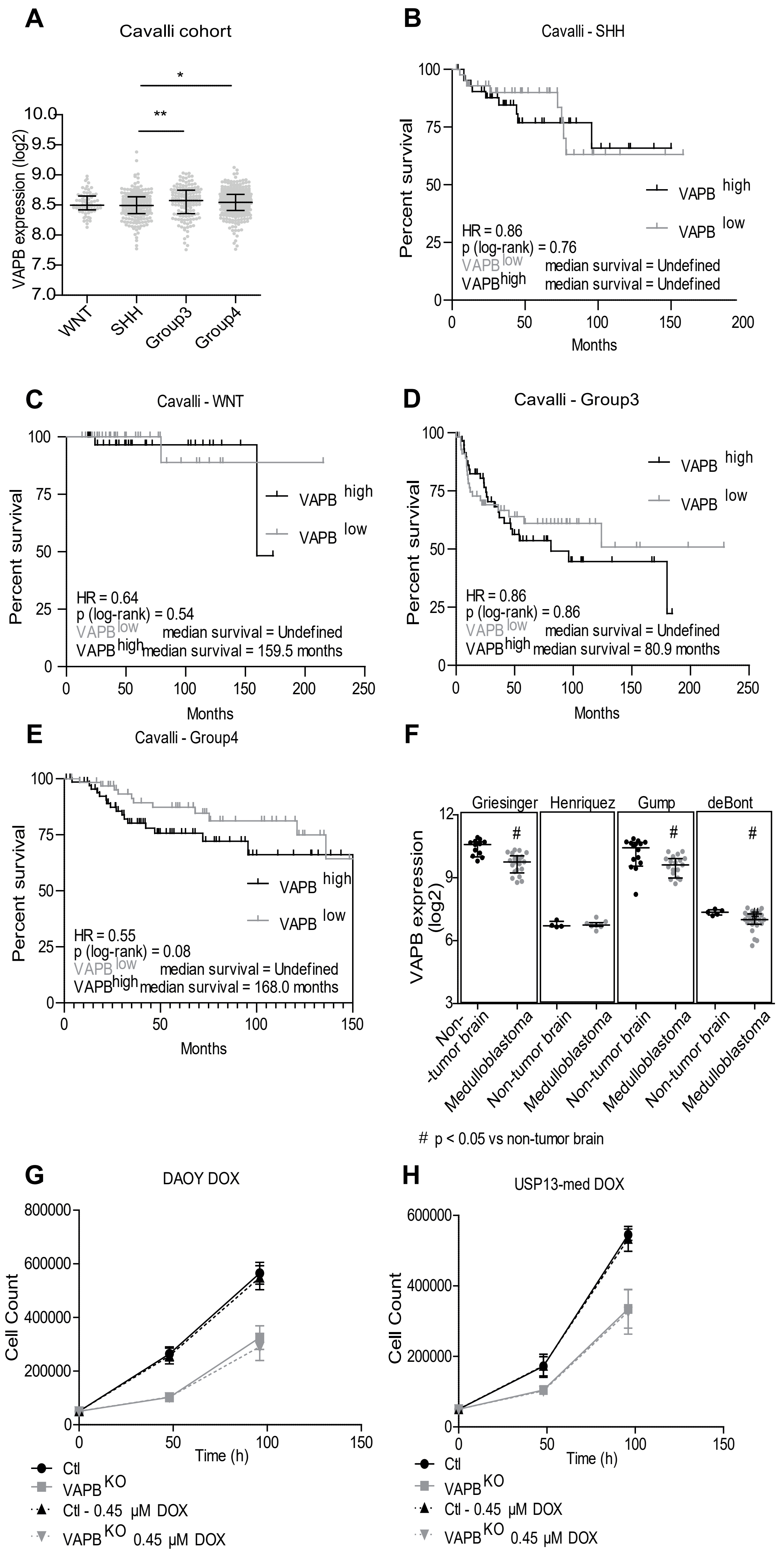
A
Cavalli cohort
VAPB expression (log2)
WNT
SHH
Group3
Group4
*
**
B
Cavalli - SHH
Percent survival
Months
VAPB high
VAPB low
HR = 0.86
p (log-rank) = 0.76
VAPB low median survival = Undefined
VAPB high median survival = Undefined
C
Cavalli - WNT
Percent survival
Months
VAPB high
VAPB low
HR = 0.64
p (log-rank) = 0.54
VAPB low median survival = Undefined
VAPB high median survival = 159.5 months
D
Cavalli - Group3
Percent survival
Months
VAPB high
VAPB low
HR = 0.86
p (log-rank) = 0.86
VAPB low median survival = Undefined
VAPB high median survival = 80.9 months
E
Cavalli - Group4
Percent survival
Months
VAPB high
VAPB low
HR = 0.55
p (log-rank) = 0.08
VAPB low median survival = Undefined
VAPB high median survival = 168.0 months
F
Griesinger
Henriquez
Gump
deBont
VAPB expression (log2)
Non--tumor brain
Medulloblastoma
Non-tumor brain
Medulloblastoma
Non-tumor brain
Medulloblastoma
Non-tumor brain
Medulloblastoma
# p < 0.05 vs non-tumor brain
G
DAOY DOX
Cell Count
Time (h)
Ctl
VAPB KO
Ctl - 0.45 μM DOX
VAPB KO 0.45 μM DOX
H
USP13-med DOX
Cell Count
Time (h)
Ctl
VAPB KO
Ctl - 0.45 μM DOX
VAPB KO 0.45 μM DOX

Supplemental. 2: VAPB expression in medulloblastoma samples. a. *VAPB* gene expression from Cavalli's dataset in different medulloblastoma subtypes. **b.** Impact of *VAPB low* or *high* expression on the survival of patients with SHH medulloblastomas in Cavalli's dataset (Log-rank test, p=0.76; n=86). **c.** Impact of *VAPB low* or *high* expression on the survival of patients with WNT medulloblastomas in Cavalli's dataset (Log-rank test, p=0.54; n=63). **d.** Impact of *VAPB low* or *high* expression on the survival of patients with G3 medulloblastomas in Cavalli's dataset (Log-rank test, p=0.86; n=114). **e.** Impact of *VAPB low* or *high* expression on the survival of patients with G4 medulloblastomas in Cavalli's dataset (Log-rank test, p=0.08; n=135). **f.** Microarray of *VAPB* gene expression from unaffected (non-tumor brain; NTB, n=50) and medulloblastoma (MED, n=77) samples were obtained from 4 cohorts and viewed by the Gliovis portal: Griesinger (NTB n=13; MED n=22), Henriquez (NTB n=16; MED n=9), Gump (NTB n=16; MED n=19) and de Bont (NTB n=5; MED n=27). **g.** Cell count of one single cell clone of DAOY control and $VAPB^{KO}$ with 0 or 0.45 µM of doxycycline at 24h and 48h. **h**. Cell count of one single cell clone of USP13-Med control and $VAPB^{KO}$ with 0 or 0.45 µM of doxycycline at 24h and 48h.

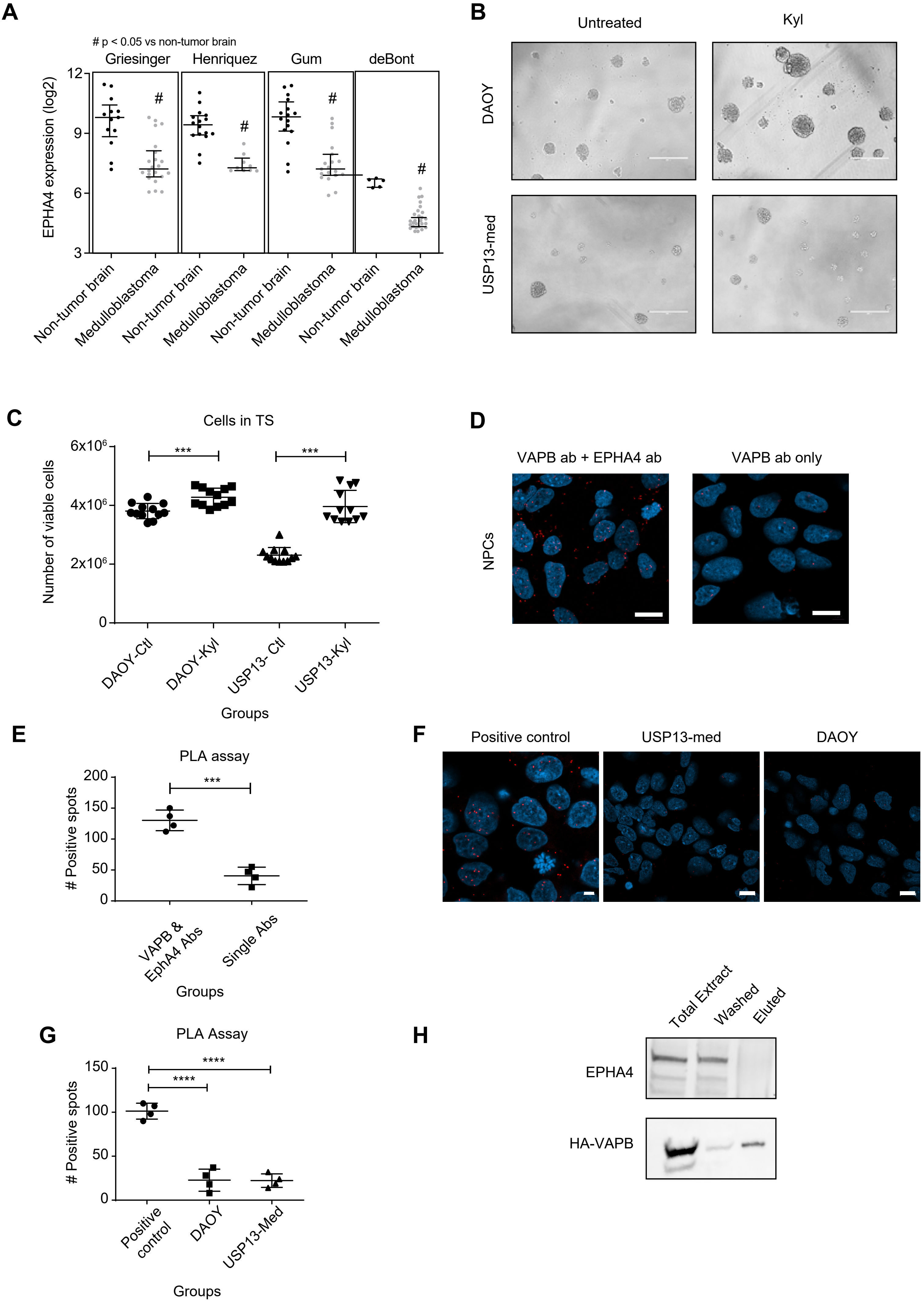
A
# p < 0.05 vs non-tumor brain
Griesinger
Henriquez
Gum
deBont
EPHA4 expression (log2)
12
9
6
3
#
Non-tumor brain
Medulloblastoma
B
Untreated
Kyl
DAOY
USP13-med
C
Cells in TS
Number of viable cells
6x10^6
4x10^6
2x10^6
0
***
DAOY-Ctl
DAOY-Kyl
USP13- Ctl
USP13-Kyl
Groups
D
VAPB ab + EPHA4 ab
VAPB ab only
NPCs
E
PLA assay
Positive spots
200
150
100
50
0
***
VAPB & EphA4 Abs
Single Abs
Groups
F
Positive control
USP13-med
DAOY
G
PLA Assay
Positive spots
150
100
50
0
****
Positive control
DAOY
USP13-Med
Groups
H
Total Extract
Washed
Eluted
EPHA4
HA-VAPB

Supplemental. 3: EPHA4 activity is decreased in medulloblastoma samples, which leads to increased CSCs sphere formation in medulloblastoma cell lines. a. *EPHA4* gene expression in primary unaffected brain and medulloblastoma samples. Microarray gene expression from unaffected (non-tumor brain; NTB, n=50) and medulloblastoma (MED, n=77) samples were obtained from 4 cohorts and viewed by the Gliovis portal: Griesinger (NTB n=13; MED n=22), Henriquez (NTB n=16; MED n=9), Gump (NTB n=16; MED n=19) and de Bont (NTB n=5; MED n=27). **b**. Representative phase contrast images of tumor spheres with or without KYL peptide144 hours post cell plating. Scale bar, 400 µm. **c.** Quantification of the number of viable cells after dissociation of tumor spheres with or without KYL peptide 144 h post cell plating (Kruskal Wallis test with Bonferroni correction; n = 7 per group). **d.** Representative images of Proximity Ligation Assay (PLA) for VAPB and EPHA4 in NPCs cell lines. Negative control was performed using only one of the antibodies. Scale bars 10 µm. **e**. Quantification of the number of dots present in the PLA for VAPB and EPHA4 in NPCs cell lines (***, $P < 0.001$, Kruskal-Wallis test n = 8 fields per group). **f**. Representative images of PLA for VAPB and EPHA4 in NPCs (positive control; scale bar 5 µm), DAOY (scale bar 10 µm) and USP13-Med (scale bar 10 µm) cell lines. **g**. Quantification of the number of dots present in the PLA for VAPB and EPHA4 in NPCs (positive control), DAOY and USP13-Med cell lines. **h.** Western blot of CO-IP total extract, washed and eluted fractions using anti-HA tag and anti-EPHA4 antibodies.

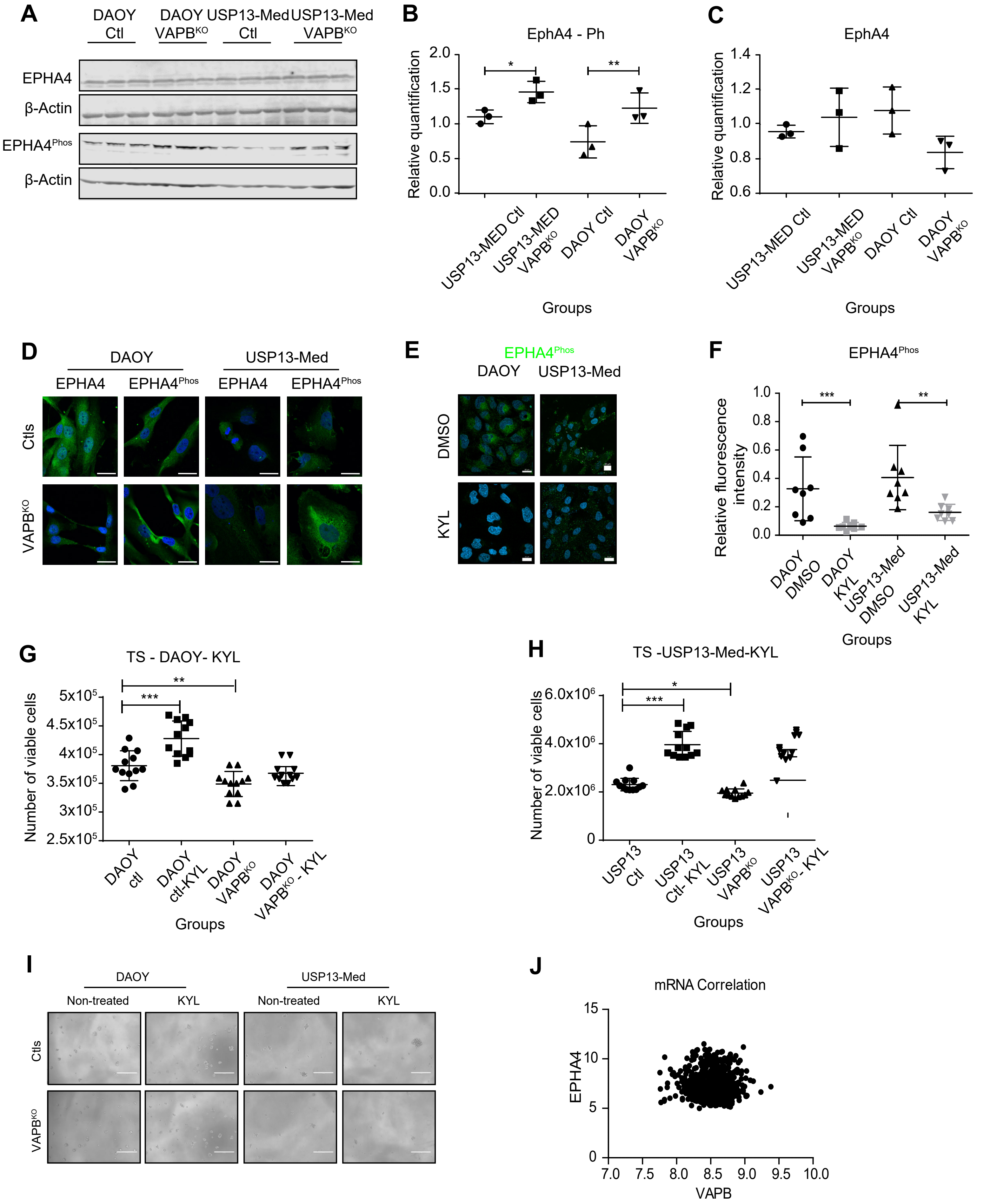

A
DAOY Ctl
DAOY VAPBKO
USP13-Med Ctl
USP13-Med VAPBKO
EPHA4
β-Actin
EPHA4Phos
β-Actin
B
EphA4 - Ph
Relative quantification
USP13-MED Ctl
USP13-MED VAPBKO
DAOY Ctl
DAOY VAPBKO
Groups
C
EphA4
Relative quantification
USP13-MED Ctl
USP13-MED VAPBKO
DAOY Ctl
DAOY VAPBKO
Groups
D
DAOY
USP13-Med
EPHA4
EPHA4Phos
Ctls
VAPBKO
E
EPHA4Phos
DAOY
USP13-Med
DMSO
KYL
F
EPHA4Phos
Relative fluorescence intensity
DAOY DMSO
DAOY KYL
USP13-Med DMSO
USP13-Med KYL
Groups
G
TS - DAOY- KYL
Number of viable cells
DAOY ctl
DAOY ctl-KYL
DAOY VAPBKO
DAOY VAPBKO - KYL
Groups
H
TS -USP13-Med-KYL
Number of viable cells
USP13 Ctl
USP13 Ctl- KYL
USP13 VAPBKO
USP13 VAPBKO- KYL
Groups
I
DAOY
USP13-Med
Non-treated
KYL
Ctls
VAPBKO
J
mRNA Correlation
EPHA4
VAPB

Supplemental 4: VAPB does not physically bind EPHA4 in medulloblastoma cells, but inhibits receptor phosphorylation. a. Western blot of DAOY and USP13-Med single cell-derived clones of $VAPB^{KO}$ and controls blotted using anti β-actin as the endogenous control, anti-total EPHA4 and anti-Tyr phosphorylated EPHA4. **b.** Quantification of the corresponding densitometry of Tyr-phosphorylated EPHA4 protein bands of DAOY and USP13-Med single cell-derived clones of $VAPB^{KO}$ and controls (unpaired t-tests, n=3). **c.** Quantification of the corresponding densitometry of total EPHA4 protein bands of DAOY and USP13-Med single cell-derived clones of $VAPB^{KO}$ and controls (unpaired t-tests, n=3). **d.** Representative images of immunofluorescence staining of total EPHA4 and Tyr-phosphorylated EPHA4 in DAOY and USP13-Med single cell-derived clones of $VAPB^{KO}$ and controls. Scale bar 20µm, **e** Representative images of immunofluorescence staining of Tyr-phosphorylated EPHA4 in DAOY and USP13-Med cell lines treated with DMSO (Vehycle) or the KYL peptide. Scale bar 20µm .**f.** Quantification of the relative fluorescence intensity of Tyr-phosphorylated EPHA4 from immunofluorescence stainings on DAOY and USP13-Med cell lines treated with DMSO (Vehycle) or the KYL peptide (Kruskal Wallis test with Bonferroni multiple comparisons corrections; n=8). **g.**. Quantification of the number of viable cells after dissociation of tumor spheres at 96 h post-cell plating of DAOY single cell-derived clones of $VAPB^{KO}$ and controls treated with vehicle or the EPHA4 phosphorylation inhibitor KYL peptide (25 µM) (Kruskal Wallis test with Bonferroni multiple comparisons corrections; n=12). **h.** Quantification of the number of viable cells after dissociation of tumor spheres at 96 h post-cell plating of USP13-Med single cell-derived clones of $VAPB^{KO}$ and controls treated with vehicle or the EPHA4 phosphorylation inhibitor KYL peptide (25 µM) (Kruskal Wallis test with Bonferroni multiple comparisons corrections; n=12). **i.** Representative phase contrast images of tumor spheres at 96h post-cell plating of DAOY and USP13-Med single cell clones of $VAPB^{KO}$ and controls treated with vehicle or the EPHA4 phosphorylation

inhibitor KYL peptide (25 μM). Scale bars represent 400 μm. **j.** Correlation between gene expression of *VAPB* and *EPHA4* in Cavalli's dataset. (n= 317, Spearman r=-0.01, p= 0.702).
