# Supplementary material for: Neurodegeneration-associated protein VAPB regulates proliferation in medulloblastoma
Source: Sci Rep. 2023 Nov 9;13:19481. doi: 10.1038/s41598-023-45319-5 (PMC10636017; doi:10.1038/s41598-023-45319-5)

Western Blots

Figure 1 A

β-Actin

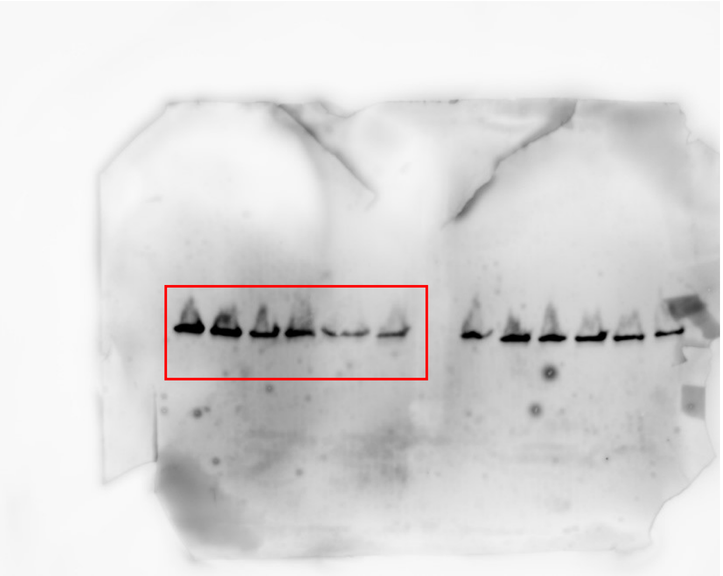

VAPB

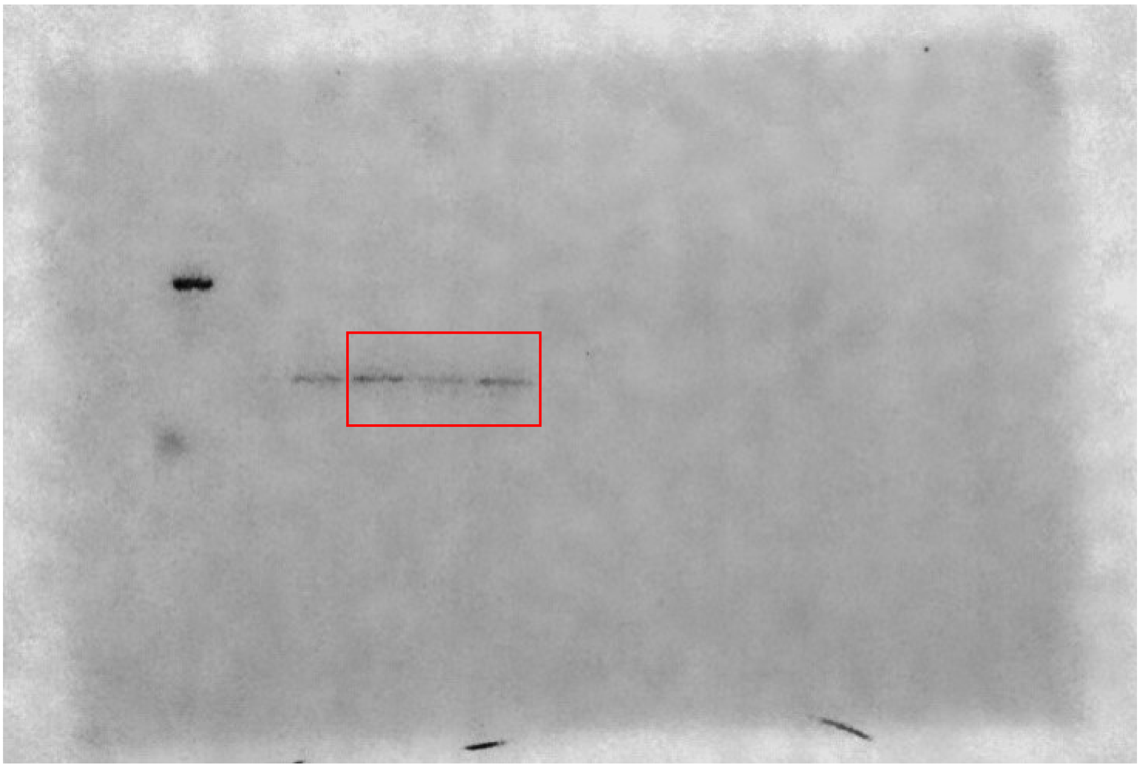

β-Actin

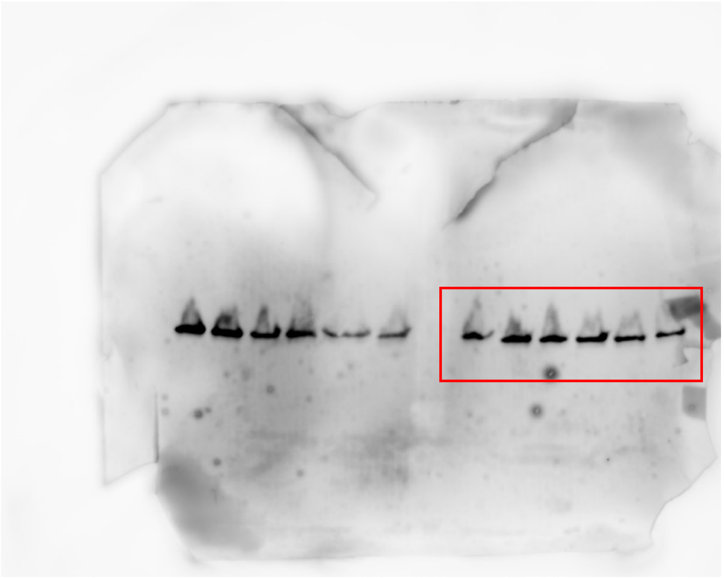

VAPB

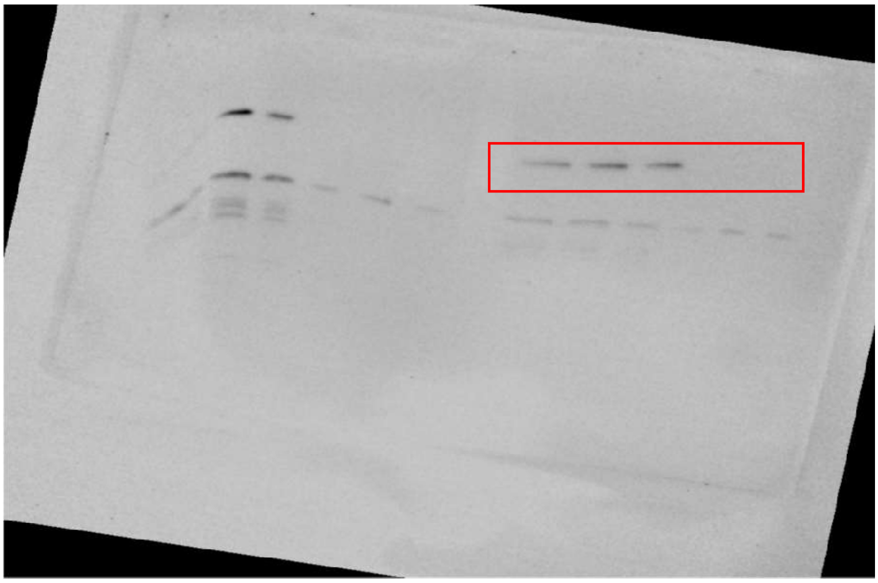

Figure 2A

β-Actin

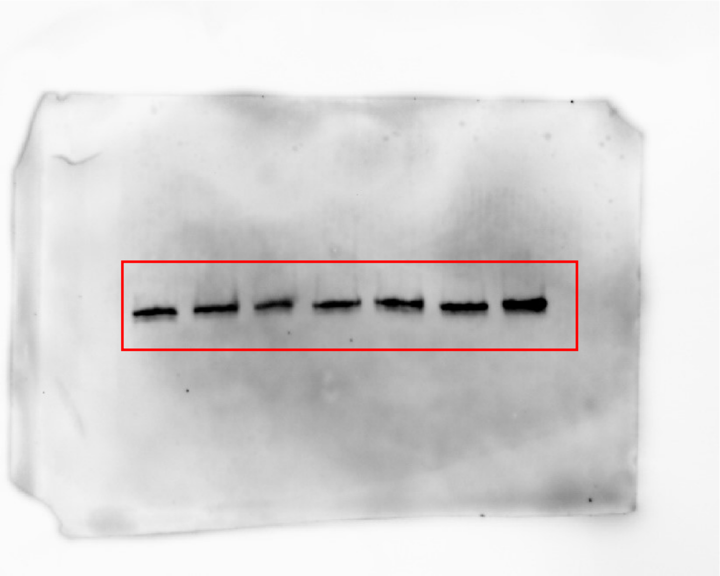

VAPB

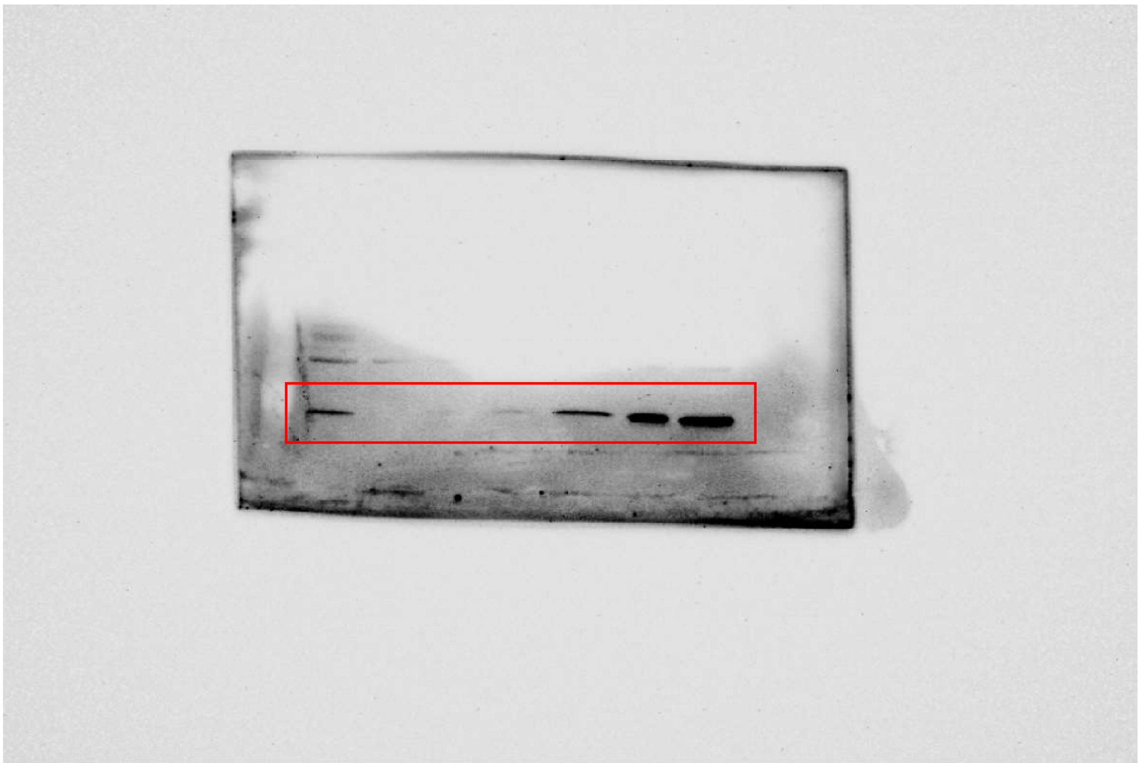

Fig 4 D

CTNNB1

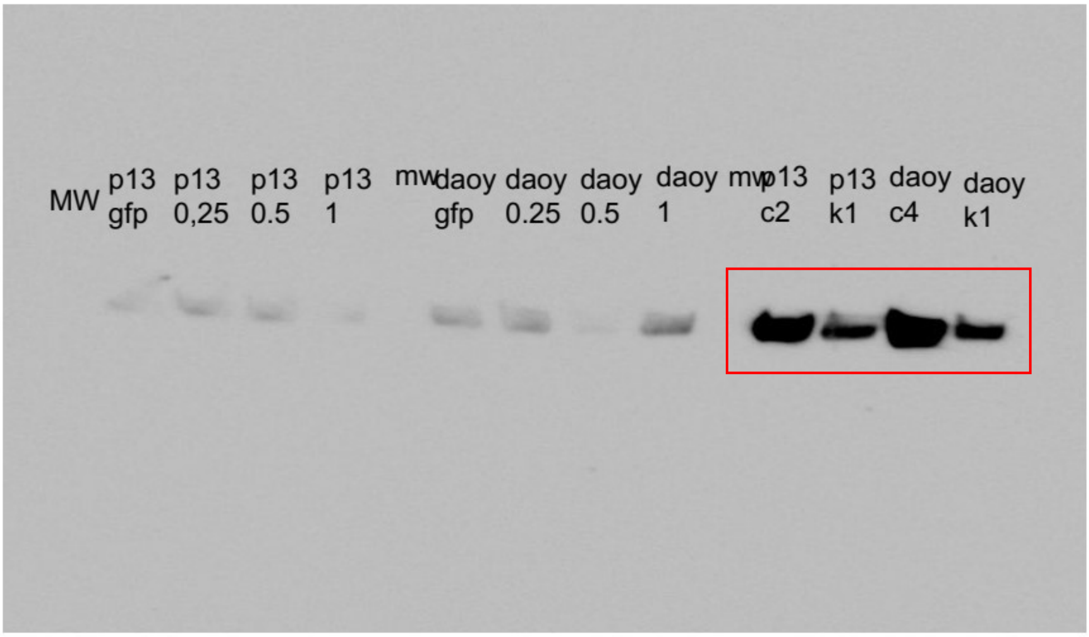

β-Actin

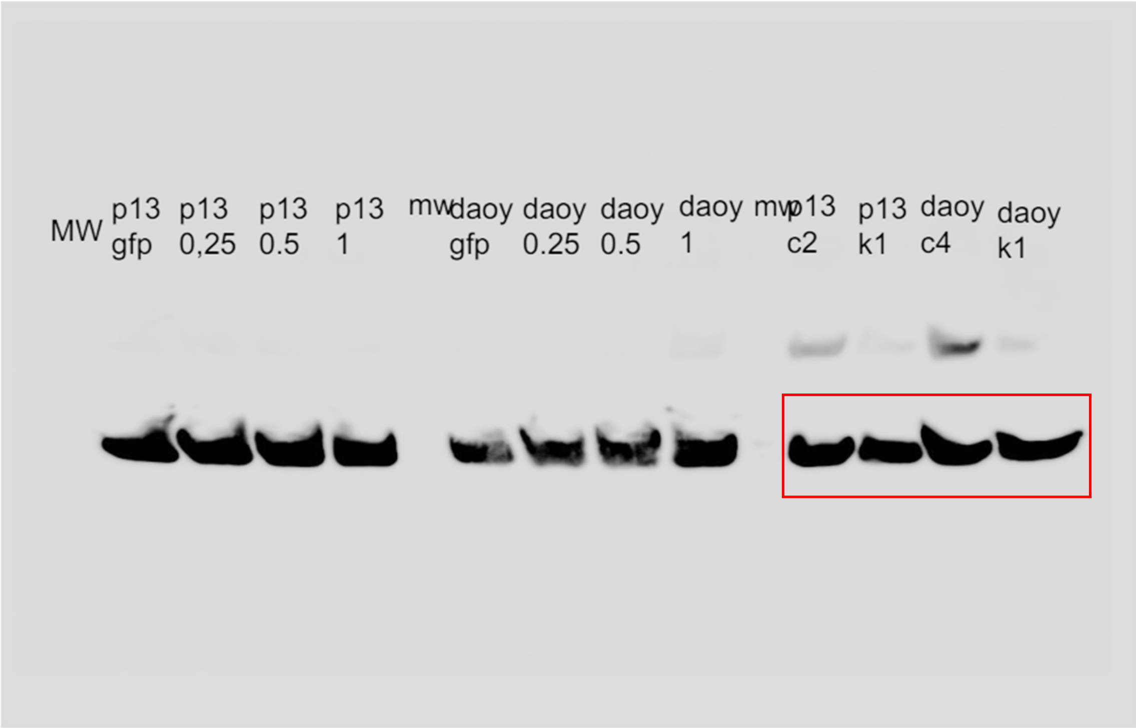

Sup 2 H

EPHA4

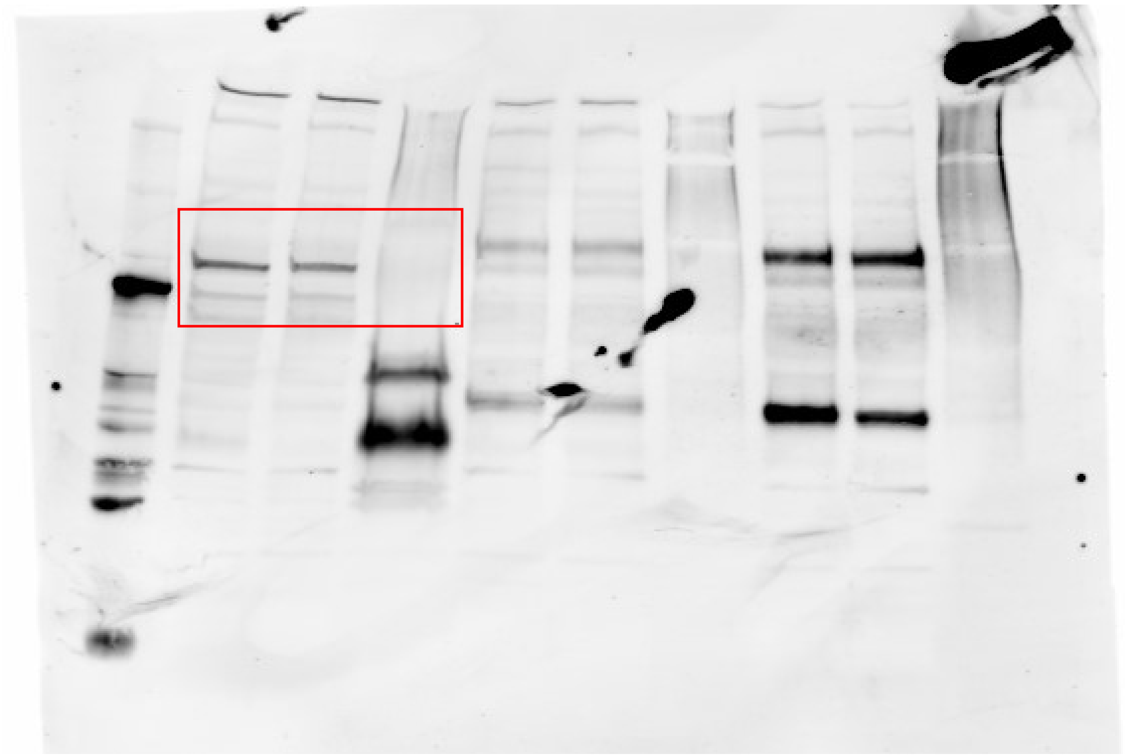

HA-VAPB

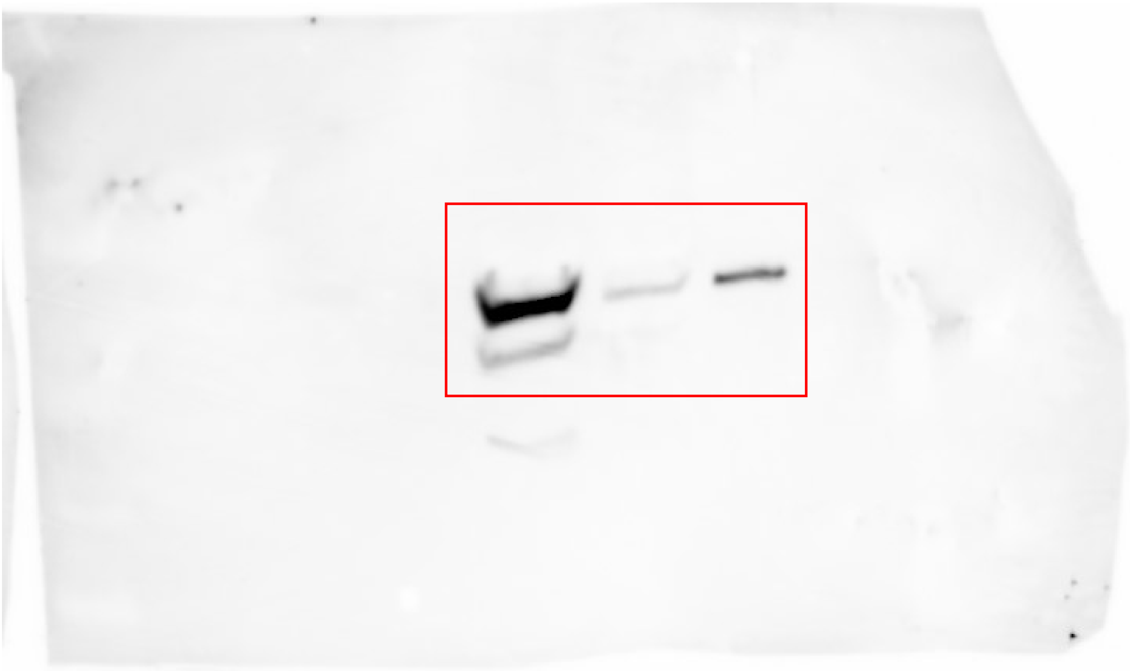

Sup 3 A

EPHA4

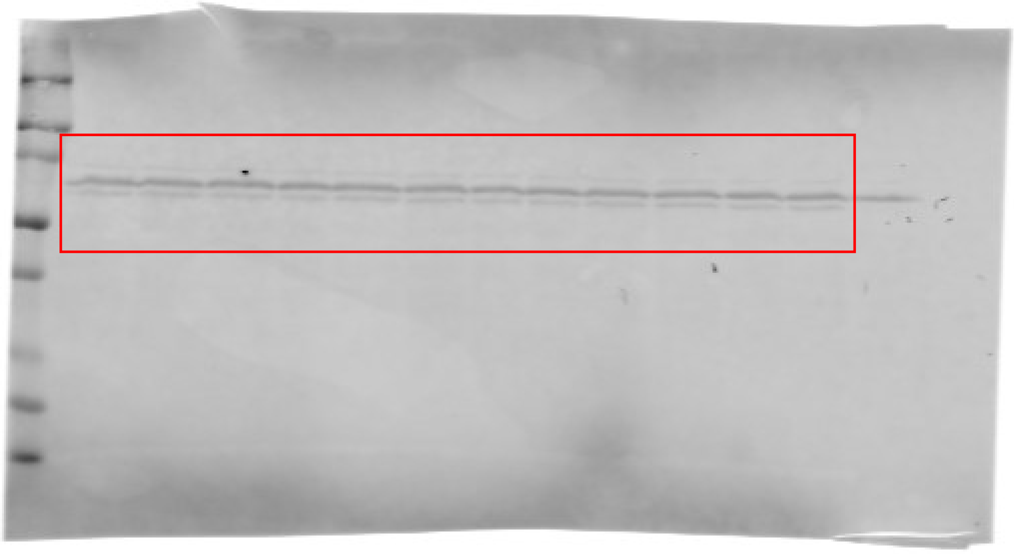

β-Actin

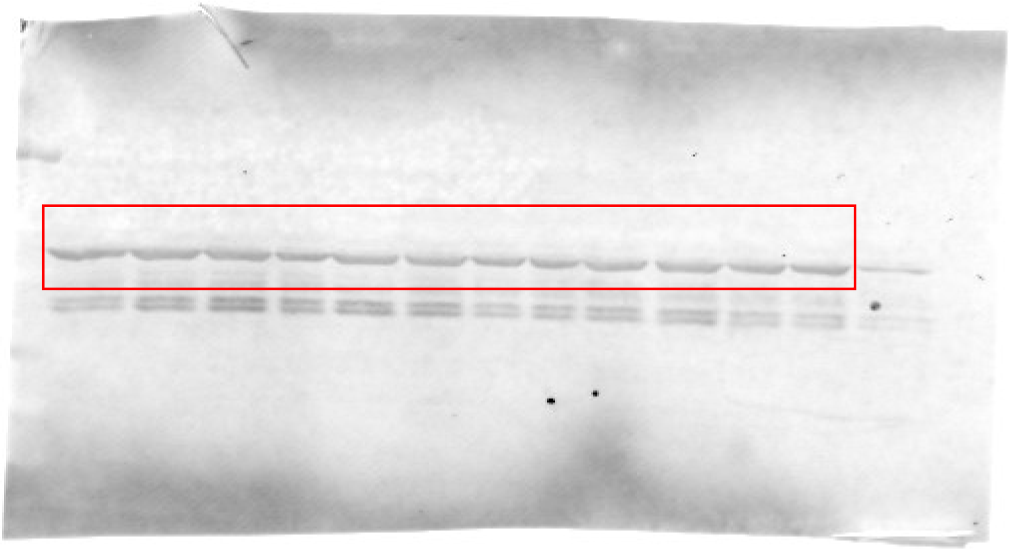

EPHA^{Phos}

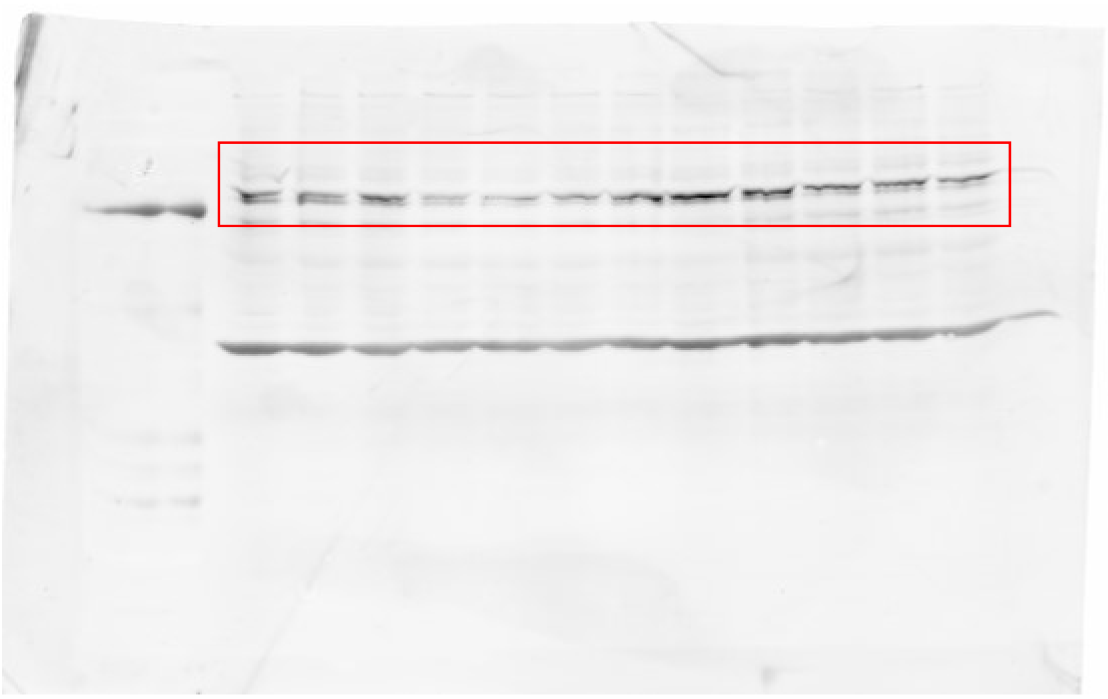

β-Actin

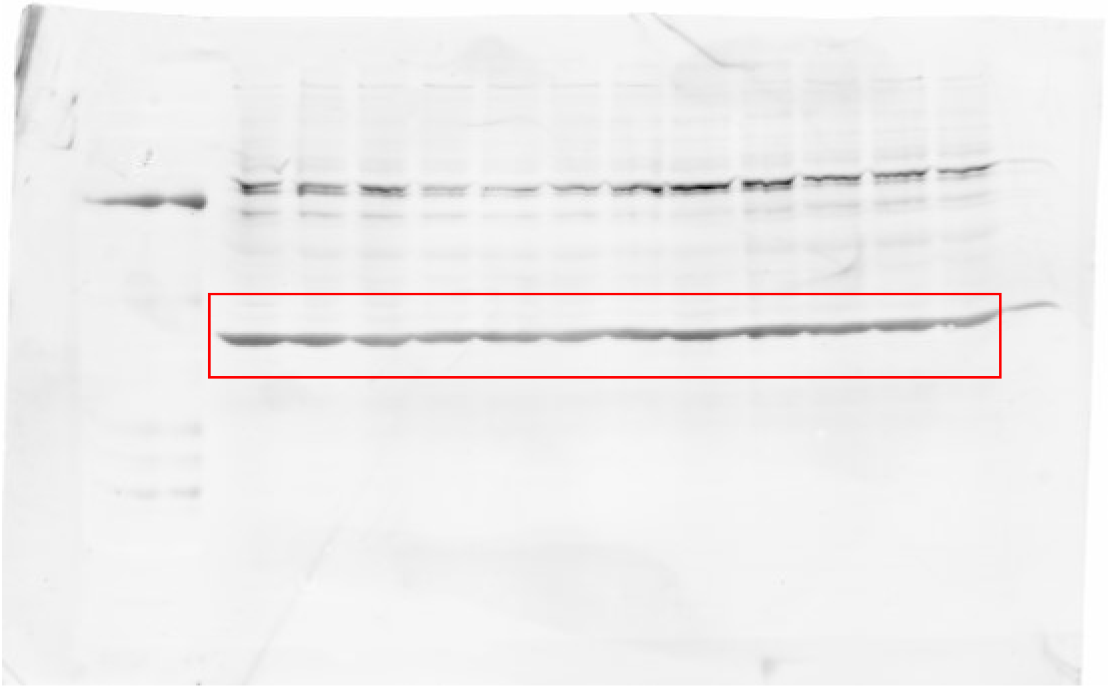

Supplement: Supplementary file 2 — Supplementary Information 2. [file 41598_2023_45319_MOESM2_ESM.pdf]
